# Transcriptomic and lipidomic analysis of the differential pathway contribution to the incorporation of erucic acid to triacylglycerol during Pennycress seed maturation

**DOI:** 10.3389/fpls.2024.1386023

**Published:** 2024-04-26

**Authors:** Ana Claver, María Ángeles Luján, José Manuel Escuín, Marion Schilling, Juliette Jouhet, María Savirón, M. Victoria López, Rafael Picorel, Carmen Jarne, Vicente L. Cebolla, Miguel Alfonso

**Affiliations:** ^1^ Department of Plant Biology, Estación Experimental Aula Dei-Consejo Superior de Investigaciones Científicas (EEAD-CSIC), Zaragoza, Spain; ^2^ Instituto de Carboquímica-Consejo Superior de Investigaciones Científicas (ICB-CSIC), Zaragoza, Spain; ^3^ Laboratoire de Physiologie Cellulaire Végétale, Univ. Grenoble Alpes, Centre National de la Recherche Scientifique-Commisariat de l'Energie Atomique-Institut National de Recherche pour l'Agriculture, l'Alimentation et l'Environnement (CNRS-CEA-INRAE), Grenoble, France; ^4^ Facultad de Ciencias, Centro de Química y Materiales de Aragón-Consejo Superior de Investigaciones Científicas (CEQMA-CSIC)-Universidad de Zaragoza, Zaragoza, Spain; ^5^ Department of Soil and Water Conservation, Estación Experimental Aula Dei-Consejo Superior de Investigaciones Científicas (EEAD-CSIC), Zaragoza, Spain; ^6^ Departamento de Química Analítica, Facultad de Veterinaria, Universidad de Zaragoza, Zaragoza, Spain

**Keywords:** *Thlaspi arvense*, seed, oil, TAG, DGAT, PDAT, VLCFAs, erucic acid

## Abstract

*Thlaspi arvense* (Pennycress) is an emerging feedstock for biofuel production because of its high seed oil content enriched in erucic acid. A transcriptomic and a lipidomic study were performed to analyze the dynamics of gene expression, glycerolipid content and acyl-group distribution during seed maturation. Genes involved in fatty acid biosynthesis were expressed at the early stages of seed maturation. Genes encoding enzymes of the Kennedy pathway like diacylglycerol acyltransferase1 (*TaDGAT1)*, lysophosphatidic acid acyltransferase (*TaLPAT)* or glycerol 3-phosphate acyltransferase (*TaGPAT)* increased their expression with maturation, coinciding with the increase in triacylglycerol species containing 22:1. Positional analysis showed that the most abundant triacylglycerol species contained 18:2 at *sn-2* position in all maturation stages, suggesting no specificity of the lysophosphatidic acid acyltransferase for very long chain fatty acids. Diacylglycerol acyltransferase2 (*TaDGAT2)* mRNA was more abundant at the initial maturation stages, coincident with the rapid incorporation of 22:1 to triacylglycerol, suggesting a coordination between Diacylglycerol acyltransferase enzymes for triacylglycerol biosynthesis. Genes encoding the phospholipid-diacylglycerol acyltransferase (*Ta*PDAT1), lysophosphatidylcholine acyltransferase (*Ta*LPCAT) or phosphatidylcholine diacylglycerolcholine phosphotransferase (*Ta*PDCT), involved in acyl-editing or phosphatidyl-choline (PC)-derived diacylglycerol (DAG) biosynthesis showed also higher expression at the early maturation stages, coinciding with a higher proportion of triacylglycerol containing C18 fatty acids. These results suggested a higher contribution of these two pathways at the early stages of seed maturation. Lipidomic analysis of the content and acyl-group distribution of diacylglycerol and phosphatidyl-choline pools was compatible with the acyl content in triacylglycerol at the different maturation stages. Our data point to a model in which a strong temporal coordination between pathways and isoforms in each pathway, both at the expression and acyl-group incorporation, contribute to high erucic triacylglycerol accumulation in Pennycress.

## Introduction

Field Pennycress (*Thlaspi arvense* L.) is a winter annual species that belongs to the Brassicaceae family. Pennycress has attracted the attention of researchers as a promising alternative oilseed feedstock for biodiesel production because of its high seed oil content and fatty acid composition. Pennycress is a prolific seed producer (Fan et al., 2013; Claver et al., 2017). Seeds contain around 29-40% oil (w/w) depending on the varieties, which is twice the amount present in other oil commodities like soybean or sunflower and very similar to that found in Camelina (Moser, 2012; Claver et al., 2017; Altendorf et al., 2019; López et al., 2021). Because of its high seed oil and fatty acid composition, enriched in erucic acid (22:1; 30-35% of total fatty acids), Pennycress oil can be used for biodiesel and biojet production with excellent properties like high cetane number, low temperature behavior and low susceptibility to oxidation when compared to other plant-oil derived biofuels (Moser et al., 2009; Moser, 2012; Fan et al., 2013). Many research efforts are being held at the agronomical level, directed towards a future crop improvement focusing on some important agronomic traits like cultivation cycle, dormancy, vernalization or seed dehiscence (Sedbrook et al., 2014; Cubins et al., 2019; López et al., 2021). At the molecular level, complete genomic sequencing (Chopra et al., 2018; McGinn et al., 2019; Geng et al., 2021; García Navarrete et al., 2022; Nunn et al., 2022) and transcriptome assembly of Pennycress genes (Dorn et al., 2013; 2015) have been reported, providing tools for its breeding. Other studies have focused in the metabolite profiling of Pennycress seed embryos (Tsogtbaatar et al., 2015; Johnston et al., 2022) or lipidomics (Romsdahl et al., 2022), providing information of lipid species accumulating in its seeds.

As a member of the Brassicaceae family, Pennycress is closely genetically related with the model plant *Arabidopsis thaliana* or to other species like *Camelina sativa* or *Brassica napus.* In Brassicaceae, Very Long Chain Fatty Acids (VLCFAs), like eicosanoic acid (20:1^Δ11^) or erucic acid (22:1^Δ13^), are present in their seed oils, although their content and distribution are highly variable among plant species. In Arabidopsis, 22:1 levels in seed lipids are very low (<2.5%), being 20:1 the major VLCFAs species, representing a 15-20% of the total fatty acids in seeds (Li-Beisson et al., 2013; Sun et al., 2013; Claver et al., 2020). In other species, such as *Brassica napus*, *Crambe abyssinica* or *Thlaspi arvense*, the total erucic acid content in the seed can range from 39 to 60% (Sun et al., 2013; Claver et al., 2020). As an example, while *T. arvense* shows a high 22:1 content, another *Thlaspideae* like *T. caerulescens* shows low 22:1 levels, similar to those from Arabidopsis (Claver et al., 2020). The reasons of this heterogeneity remain unclear. Our group recently performed a functional characterization of the Pennycress *Ta*FAE1 elongase (Claver et al., 2020), responsible of the biosynthesis of erucic acid in the endoplasmic reticulum (ER) through the sequential elongation of C18 acyl-CoA substrates to produce 20:1-CoA and 22:1-CoA (Ghanevati and Jaworski, 2001; Katavic et al., 2001). The complementation of a series of Arabidopsis mutant lines with the Pennycress *TaFAE1* gene indicated that the elongase from Pennycress showed higher affinity to 20:1-CoA than the Arabidopsis one, suggesting that different enzyme affinities might explain the different erucic acid content in their seed oil (Claver et al., 2020). In fact, differences in substrate affinity have been reported for many enzymes of the seed oil biosynthetic pathway in different plant species (Lager et al., 2013; Aznar-Moreno et al., 2015; Demski et al., 2019) but, with the exception of the FAE1 elongase (Claver et al., 2020), this has not been analyzed into detail in Pennycress.

Triacylglycerol (TAG) is the major fraction in plant seed oils, representing an 80-90% of total seed lipids (Bates and Browse, 2012). In Pennycress seeds, TAG was the major reservoir of erucic acid increasing during seed maturation as reported previously by our group in a thin layer chromatography-gas chromatography (TLC-GC) study (Claver et al., 2017) or, more recently, in a lipidomic analysis (Romsdahl et al., 2022). Different pathways contribute to TAG biosynthesis in the seed. On one hand, TAG biosynthesis is performed by a series of enzymes (glycerol 3-phosphate acyltransferase, GPAT; lysophosphatidic acid acyltransferase, LPAT; phosphatidic acid phosphatase, PAP; and acyl-CoA:diacylglycerol acyltransferase, DGAT), that perform the sequential acylation of the *sn-1*, *sn-2* and *sn-3* positions of the glycerol backbone through the Kennedy pathway (Ohlrroge and Browse, 1995). DGAT enzymes are responsible of the final acylation to produce TAG, whose activity has been shown to determine the carbon flow into TAG (Weselake et al., 2009; Li et al., 2010; Bates and Browse, 2012; Bates et al., 2013). Another pathway for TAG biosynthesis is the acyl editing pathway. Acyl editing is a deacylation-reacylation cycle in which an acyl group from phosphatidyl choline (PC) is released to the acyl-CoA pool, generating lyso-PC by the reverse action of an acyl-CoA:lyso-phosphatidylcholine acyltransferase (LPCAT) or a phospholipase A (Stymne and Stobart, 1984; Chen et al., 2011). Re-esterification of lyso-PC by LPCAT generates PC, leading to no modification of the PC content. Through this pathway, modified fatty acids, mainly polyunsaturated fatty acids (PUFAs), can enter the acyl-CoA pool for glycerolipid biosynthesis (Bates et al., 2009; reviewed in Bates and Browse, 2012). In addition, a direct transfer of an acyl group from the *sn-2* position of PC to the *sn-3* hydroxyl of diacylglycerol (DAG) producing TAG occurs by the action of the phospholipid:diacylglycerol acyltransferase (PDAT; Dahlqvist et al., 2000). The lyso-PC generated by PDAT can be reacylated to PC by LPCAT through the acyl editing cycle (Bates and Browse, 2012; Wang et al., 2012; Xu et al., 2012; Bates et al., 2013). PC-derived DAG interconversion by phosphatidylcholine diacylglycerolcholine phosphotransferase (PDCT) is another pathway of DAG supply for TAG synthesis (Bates and Browse, 2012; Wang et al., 2012; Bates et al., 2013). The contribution of these different TAG biosynthetic pathways may vary among species. Thus, in Arabidopsis, 40% of the PUFAs found in TAG are believed to be incorporated through the acyl editing pathway (Lu et al., 2009). On the contrary, in *Crambe abyssinica*, a high erucic acid containing species, *sn-1* and *sn-3* positions of TAG used acyl groups incorporated outside the acyl editing pathway, suggesting a major role of DGAT enzymes (Guan et al., 2014). Besides this contribution of the Kennedy pathway, significant PDAT activity (10% of total DGAT one) was detected in Crambe seeds in periods of rapid seed oil accumulation, indicating a specific contribution of the acyl-editing pathway to TAG biosynthesis in this erucic containing species (Furmanek et al., 2014). In fact, the incorporation of VLCFA to TAG through both pathways has been pointed out as a possible bottleneck responsible of the different erucic acid content in plant seed oils (Guan et al., 2014). However, the specific contribution of these different TAG biosynthetic pathways for the incorporation of 22:1 as well as other acyl groups in Pennycress is still unknown.

In this work, we have performed a transcriptional study of the whole seed maturation process in an attempt to analyze the expression patterns of genes encoding enzymes of the Kennedy, acyl editing and PC-derived DAG/TAG biosynthetic pathways, studying their temporal regulation and their specific contribution to TAG biosynthesis at different seed maturation stages. In parallel, lipidomic analysis was performed to characterize the lipid species and acyl group distribution during Pennycress seed maturation. RNA-Seq and qPCR analysis of genes involved in fatty acid and TAG biosynthesis showed a complex regulation in which genes encoding enzymes belonging to the different TAG biosynthetic pathways were expressed in a concerted manner with differences in their expression profiles between the earlier and the latter stages of seed maturation. The lipidomic analysis showed a higher incorporation of VLCFAs like 20:1 and particularly 22:1 to TAG at the intermediate-latter stages, coinciding with the higher TAG accumulation in the seed, although TAG species containing 22:1 were already detected at the earlier ones. Liquid chromatography-mass spectrometry (LC-MS) analysis of the rest of the lipid classes present in the total lipid fractions provided information about the lipid reservoirs of 22:1 for its incorporation to TAG. Positional analysis was also performed to analyze the specificity of the TAG biosynthetic enzymes for the incorporation of specific acyl groups to the different positions in TAG. Our data point to a strong temporal regulation during seed maturation of the expression of genes involved in TAG biosynthesis as well as glycerolipid and acyl group distribution, suggesting a different contribution of the different pathways for TAG biosynthesis and for the incorporation of 22:1 to TAG in the Pennycress seed.

## Materials and methods

### Plant materials

**Figure 1:**
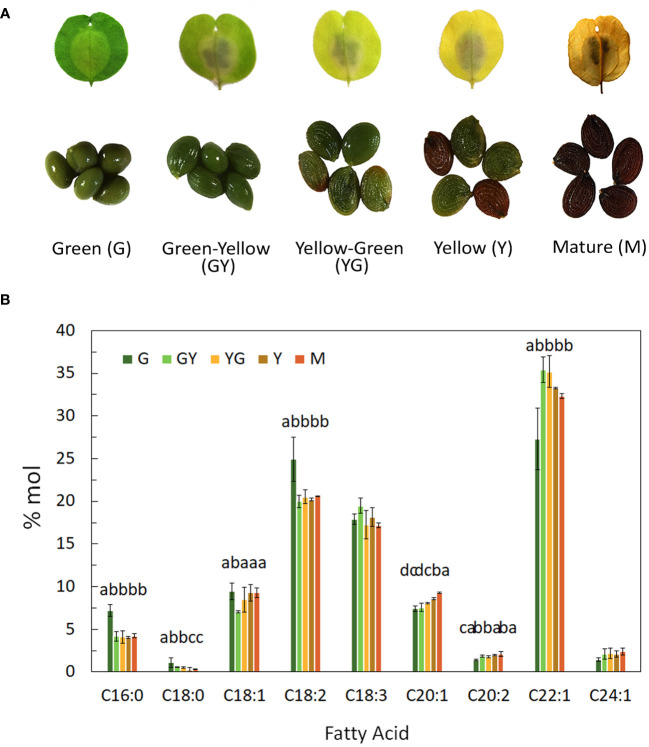
Fatty acid composition content during Pennycress seed maturation. **(A)** Photograph showing each of the five maturation stages used in this study including photographs of the seeds in each stage, **(B)** Fatty acid composition from total lipids extracted from the different stages of seed maturation; G, green seed, GY, green-yellow seed, YG, yellow-green seed, Y, yellow seed, M, mature seed. Seeds were pooled for each stage and data were obtained from three independent biological replicates. Data represent means ± SD. Different letters above the bars indicate significant differences among the different seed maturation stages for each fatty acid (P< 0.05).

Pennycress (*Thlaspi arvense* L.) seeds from the SPRING32 germline were used in this study. These seeds were obtained from the Nottingham Arabidopsis Stock Centre (NASC), UK. Seeds were germinated in plates on wet Whatman paper without addition of any other supplement. For germination, seeds were vernalized for 3 days at 4°C and then moved to a growth chamber for additional 10-14 days. No vernalization treatment was required for fully development of flowers and seeds in this germline. Once germinated, seeds were transferred to pots containing a 75:25 mixture of substrate (peat moss, Kekkilä White 420W: vermiculite) and grown in a bioclimatic chamber under a light intensity of 120-150 μmol m^-2^ s^-1^, with a 16h/8h light/dark photoperiod at 22°C and a relative humidity of 45%. For seed maturation studies, seeds from five developmental stages corresponding to GREEN (G, 12 days after flowering, DAF), GREENYELLOW (GY, 19 DAF), YELLOWGREEN (YG, 26 DAF), YELLOW (Y, 33DAF) and MATURE (M, 45 DAF), were chosen for analysis, similarly as described in Claver et al. (2017). Seeds separated from the pods, corresponding to these different maturation stages (**Figure 1A**), were harvested, frozen in liquid nitrogen, and stored at -80°C for further analysis unless indicated otherwise.

### RNA isolation, cDNA synthesis and qPCR expression analysis

Total RNA was isolated from 0.1 g of *Thlaspi arvense* seeds from the five maturation stages analyzed using the Cethyl Trimethyl Ammonium Bromide (CTAB)-LiCl extraction method of Gasic et al. (2004). RNA concentration and integrity were measured in a Nanodrop 2000 UV-Vis Spectrophotometer (Thermo Scientific). cDNAs were synthesized from 3 µg of total RNA using SuperScript III Reverse Transcriptase (Fischer) and oligo dT primer, according to the manufacturer’s instructions. Quantitative PCR (qRT-PCR) of target genes was performed using a 7500 Real Time PCR System (Applied Biosystems), SYBR Green Master Mix (Applied Biosystems) and specific primers (**Supplementary Table S1**). The Ct values were calculated relative to *ACT2* and *EF1α* reference genes using 2^-ΔΔCt^ method (Livak and Schmittgen, 2001). Data were obtained from the analysis of at least three biological samples with three independent technical repeats for each sample.

### RNA-Seq analysis

RNA-Seq libraries were prepared and sequenced on an Illumina NovaSeq6000 at Novogene Ltd (www.novogene.uk). Ten libraries, corresponding to two biological replicates of the five different seed developmental stages, were constructed in this work. Messenger RNA was purified from total RNA using poly-T oligo-attached magnetic beads. The first strand cDNA was synthesized using random hexamer primers, followed by the second strand cDNA synthesis using either dUTP for directional library or dTTP for non-directional library. The library was checked with Qubit and real-time PCR for quantification and bioanalyzer for size distribution detection. Quantified libraries were pooled and sequenced. Original image data file from high-throughput sequencing was transformed to sequenced reads by CASAVA. Raw data were stored in FASTQ(fq) files, containing sequences of reads and corresponding base quality. For each library, raw reads, clean reads, quality parameters as Q20 (%), Q30 (%) and QC (%), as well as the mapped percentage were first monitored. The results are available in **Supplementary Table S2**. Once raw reads were cleaned, alignments were performed with HISAT2 (Mortazavi et al., 2008). Mapped regions were classified as exons, introns, or intergenic regions, and annotated with respect to the Pennycress reference genome (www.ncbi.nl,.gov/assembly/GCA_91186555.2; Nunn et al., 2022). A 79.97% of the clean reads were detected in exonic regions, while 2.71% and 17.30% were detected in intronic and intergenic regions, respectively. The quality of the data was tested through a Pearson correlation analysis, showing that all libraries from the biological replicates were highly related and, therefore, good for the gene expression analysis. Gene expression level was estimated by FPKM values (short for the expected number of Fragments Per Kilobase of transcript sequence per Millions base pairs sequenced; Mortazavi et al., 2008). Correlation of the gene expression levels between samples was estimated by Pearson coefficient greater than 0.92 and the R^2^ greater than 0.8. Up-regulated and down-regulated genes were identified for each seed maturation stage comparison. The screening criteria used for differential expressed genes was log_2_(FoldChange) ≥ 1, and padj ≤ 0.05. Similar expression patterns were clustered together using the FPKM values of genes. The overall results of FPKM cluster analysis, clustered using the log_2_(FPKM + 1) value, were generated. When required, heatmaps were generated using the online tool https://bar.utoronto.ca/ntools/cgi-bin/ntools_heatmapper_plus.cgi, using the log_2_ ratio of (FPKM + 1) in each sample pair. We used the clusterProfiler (Yu et al., 2012) software for enrichment analysis, including GO Enrichment, DO Enrichment, KEGG and Reactome database Enrichment. GO terms with padj < 0.05 were regarded as significant enrichment. In the results of the GO enrichment analysis, the most significant 30 Terms were selected for display. The different colors represent the three GO subclasses of biological process (BP), cellular component (CC), and molecular function (MF). KEGG pathways with padj < 0.05 were regarded as significant enrichment. In the KEGG enrichment results, the most significant 20 KEGG pathways were selected for display.

### Lipid and fatty acid composition analysis

Total lipids were extracted from Pennycress seeds (0.1 g) with chloroform:methanol (2:1, v:v) as described by Bligh and Dyer (1959). For total fatty acid quantification, we followed the method from Li et al. (2006) through direct whole seed transmethylation using triheptadecanoin (30-35 μg) as internal standard. Fatty acid methyl esters of total lipids were analyzed by GC-FID as described in Claver et al. (2017).

### LC-MS analysis

Quantification of each lipid species was carried out on the LIPANG platform by liquid chromatography-MS/MS as previously described (Jouhet et al., 2017). The lipid extracts corresponding to 25 nmol of total fatty acids were dissolved in 100 µL of chloroform/methanol [2/1, (v/v)] containing 125 pmol of each internal standard. Internal standards used were Phosphatidylethanolamine (PE) 18:0-18:0 and DAG 18:0-22:6 from Avanti Polar Lipid and Sulfoquinovosyl diacylglycerol (SQDG) 16:0-18:0 extracted from spinach thylakoid (Demé et al., 2014) and hydrogenated as described in Buseman et al. (2006). Lipids were then separated by high Performance Liquid Chromatography (HPLC) and quantified by MS/MS.

The HPLC separation method was adapted from Rainteau et al. (2012). Lipid classes were separated using an Agilent 1260 Infinity II HPLC system using a 150 mm×3 mm (length × internal diameter) 5 µm diol column (Macherey-Nagel), at 40°C. The mobile phases consisted of hexane/isopropanol/water/ammonium acetate 1M, pH5.3 [625/350/24/1, (v/v/v/v)] (A) and isopropanol/water/ammonium acetate 1M, pH5.3 [850/149/1, (v/v/v)] (B). The injection volume was 20 µL. After 5 min, the percentage of B was increased linearly from 0% to 100% in 30 min and stayed at 100% for 15 min. This elution sequence was followed by a return to 100% A in 5 min and equilibration for 20 min with 100% A before the next injection, leading to a total runtime of 70 min. The flow rate of the mobile phase was 200 µL/min. The distinct glycerolipid classes were eluted successively as a function of the polar head group.

Mass spectrometric analysis was done on a 6470 triple quadrupole mass spectrometer (Agilent) equipped with a Jet stream electrospray ion source under following settings: Drying gas heater: 260°C, Drying gas flow 13 L/min, Sheath gas heater: 300°C, Sheath gas flow: 11L/min, Nebulizer pressure: 25 psi, Capillary voltage: ± 5000 V, Nozzle voltage ± 1000. Nitrogen was used as collision gas. The quadrupoles Q1 and Q3 were operated at widest and unit resolution respectively. PC analysis was carried out in positive ion mode by scanning for precursors of m/z 184 at a collision energy (CE) of 35 eV. SQDG analysis was carried out in negative ion mode by scanning for precursors of m/z -225 at a CE of -55V. PE, phosphatidylinositol (PI), phosphatidylserine (PS), phosphatidylglycerol (PG), Phosphatidic acid (PA), monogalactosil diacylglycerol (MGDG), and digalactosil diacylglycerol DGDG measurements were performed in positive ion mode by scanning for neutral losses of 141 Da, 277 Da, 185 Da, 189 Da, 115 Da, 179 Da, and 341 Da at CEs of 29 eV, 21eV, 21 eV, 25 eV, 25 eV, 8 eV and 11 eV, respectively. Quantification was done by multiple reaction monitoring (MRM) with 30 ms dwell time. DAG and TAG species were identified and quantified by MRM as singly charged ions [M+NH_4_]^+^ at a CE of 19 and 26 eV respectively with 30 ms dwell time. CL species were quantified by MRM as singly charged ions [M-H]- at a CE of -45 eV with 50 ms dwell time. The list of MRM transition was adapted from Romsdahl et al., 2022, and presented in **Supplementary Table S3**. Mass spectra were processed by MassHunter Workstation software (Agilent) for identification and quantification of lipids. Lipid amounts (pmol) were corrected for response differences between internal standards and endogenous lipids and by comparison with a quality control (QC). QC extract corresponds to a known lipid extract from arabidopsis cell culture qualified and quantified by TLC and GC-FID as described by Jouhet et al. (2017).

### High-performance thin-layer chromatography-densitometry-tandem mass spectrometry analysis

Instruments for sample application, chromatographic development, densitometry and HPTLC-MS coupling were from CAMAG (Muttenz, Switzerland).

### Plate pre-conditioning

Before being used, plates were immersed in tetrahydofuran (THF) for cleaning by diffusion. Subsequently, they were dried at 70° C and vacuum (50 mbar) for 15 min. Clean plates provided stable baselines, monitored by UV at 190 nm. To avoid possible impurities from the solvent itself being deposited uniformly on the plate, an additional pre-development with the chosen mobile phase was carried out, in the absence of sample, up to 90 mm migration distance (m.d.).

### Standards and sample application

Standards and chemicals used in the analysis are listed in **Supplementary Data Information**. Solutions of each above individual standards were also applied in triplicate on the same plate (concentration: 0.33-2 mg/ml per standard in DCM : MeOH, (1:1 v:v); application of effective mass: 3 μg/band). In order to optimize the applied sample volume and thus save sample, the ATS4 filling quality method was used. In a given plate, minimal distance between tracks was 6 mm and distances from the lateral and lower plate edges were 10 mm. One or more tracks were left empty, as blanks. The five seed maturation stages were studied: Two samples (lipid extracts) per maturation stage, corresponding to two different batches, were analyzed by HPTLC-densitometry-tandem MS. Each sample was applied on three different plates (9 measurements per sample). Samples were dissolved (3-4 mg ml^-1^) in DCM : MeOH (1:1 v:v). 4 μl/band were applied on the corresponding HPTLC silica gel plate, at least in triplicate, as 4-mm bands, by using the Automatic TLC Sampler (ATS4) system.

### Chromatographic development and densitometric detection

Isocratic chromatographic development up to 70 mm-migration distance was performed in a horizontal developing chamber (20 x 10 cm) using an acidic medium: *n*-heptane (C7), methyl t-butyl ether (MTBE) and acetic acid (AcH) (70:30:1, v:v:v). The selected isocratic development applied to a standard mixture allowed to separate at baseline most of neutral lipid families in samples, e.g. mono-(MAG), di- (DAG), tri-acylglycerides (TAG), fatty acids (FA), fatty acyl-(FAE) and cholesteryl esters (ChOE), and phosphatidylcholine (PC, at the application point), over a total m.d. of 70 mm. Three plates per lipid extract sample of each maturation stage were developed on different days. Detection was carried out using a TLC Scanner 3 densitometer in mode UV at 190 nm. Baseline of chromatograms was corrected manually. WinCATS software (v 1.4.3.6336) was used to control and process data from sample application, chromatography and densitometry. HPTLC separation was first tested on silica gel plates using the standards mentioned in Materials and Methods. Chromatograms related to standards are shown in **Supplementary Figure S1**. HPTLC chromatograms corresponding to samples at the different maturation stages, detected at UV 190 nm. Development conditions were selected to clearly separate TAG (55 mm, m.d.) from the other neutral lipid families (**Supplementary Figure S2**).

### Coupling with tandem mass spectrometry

TLC-MS Interface 2 was used from an extraction of each TAG peak directly from the plate. A detail of elution-based interface description and operation can be found elsewhere (Sancho-Albero et al., 2022). It was equipped with an oval, 4 x 2-mm extraction head that was positioned on the corresponding TAG-band maximum, whose the x,y coordinates were provided by WinCats software, using a laser crosshair. Then the interface head was lowered. MeOH was delivered for band extraction at 0.2 mL/min by using a PU-2080 HPLC pump (Jasco, Tokyo, Japan). The eluate was directed through a 2-μm stainless steel frit to remove silica gel and then sent to the mass spectrometer. Electrospray ESI-MS in positive mode (ESI^+^) was selected and mass spectra were registered on an Ion trap Amazon Speed Spectrometer (Brüker Daltonics, Bremen, Germany). ESI^+^-MS was conducted with capillary and endplate offset voltages of -4500 and -500 V, 36 psi as pressure of the nebulizer gas (N_2_), 6.0 L/min as flow rate of the drying gas (N_2_) and 120°C as drying gas temperature. Spectra were acquired in the m/z 70–1500 range at the ultra-scan mode. Bruker Daltonics Trap Control software packages v 8.0 and Data Analysis v 5.2 were used to control the mass spectrometer and process data. For each TAG peak, several HPTLC- ESI^+^-MS experiments were performed from replicate bands and confirmation of identity was carried out by MS^2^. These experiments were performed from different plates. The HPTLC-ESI^+^-MS operating conditions are specified for each case in the Results and Discussion section.

MS acquisition was performed by a Quadrupole Time-of-Flight (QTOF) mass spectrometer equipped with an Electrospray Ionization Source (ESI) (MicrOTOF-Q, Bruker Daltonics, Bremen, Germany). High Resolution (HR)-MS experiments were carried out in positive ion mode. The nebulizer gas (N2) pressure, the drying gas (N2) flow rate and the drying gas temperature were 1.6 bar, 8.0 L/min, and 190°C, respectively. Spectra were acquired in the m/z 50–2000 range. The mass axis was calibrated by using Na-formate adducts [10 mmol/l NaOH, 2.5% (v/v) formic acid and 50% (v/v) 2-propanol] that were introduced through a divert valve at the beginning of each direct injection. Bruker Daltonik software packages micrOTOF Control v.3.4 and HyStar v.3.2 were used to control the system. Data Analysis v.4.2 was used to process the data.

### Statistical analysis

Data are expressed as means ± SD, with at least three replicates in each experimental group. The statistical comparisons among the different developmental stages during seed maturation of Pennycress were made using one-way analysis of variance (ANOVA) and means were compared with the Duncan’s multiple range test (*P* < 0.05). When data showed non-normality, log or reciprocal transformations were made and ANOVA conducted with the transformed data.

## Results

### Maturation patterns in developing Pennycress seeds

Seeds from five different developmental stages of Pennycress SPRING32 germline, from the youngest GREEN (G) stage to the final MATURE (M) stage, were analyzed in this study (**Figure 1A**). These five stages covered the whole seed maturation process. Temporal changes in fatty acid composition were analyzed in total lipid fractions from each developmental stage. Erucic acid was highly abundant in all stages (**Figure 1B**). 22:1 represented a 20% of total fatty acids at the G initial stage, increasing from the G to the GY and YG stages, reaching values higher to 35%, to then slowly decrease in the latter Y and M stages (**Figure 1B**). The lower 22:1 values at the younger G stage were concomitant with higher 18:2 levels, that later decreased upon Pennycress seed maturation (**Figure 1B**). Other VLCFAs like 20:1 and 24:1 were also detected in all seed maturation stages, increasing their levels during the whole seed maturation process (**Figure 1B**).

### Differential gene expression at the different stages of Pennycress seed maturation

**Figure 2:**
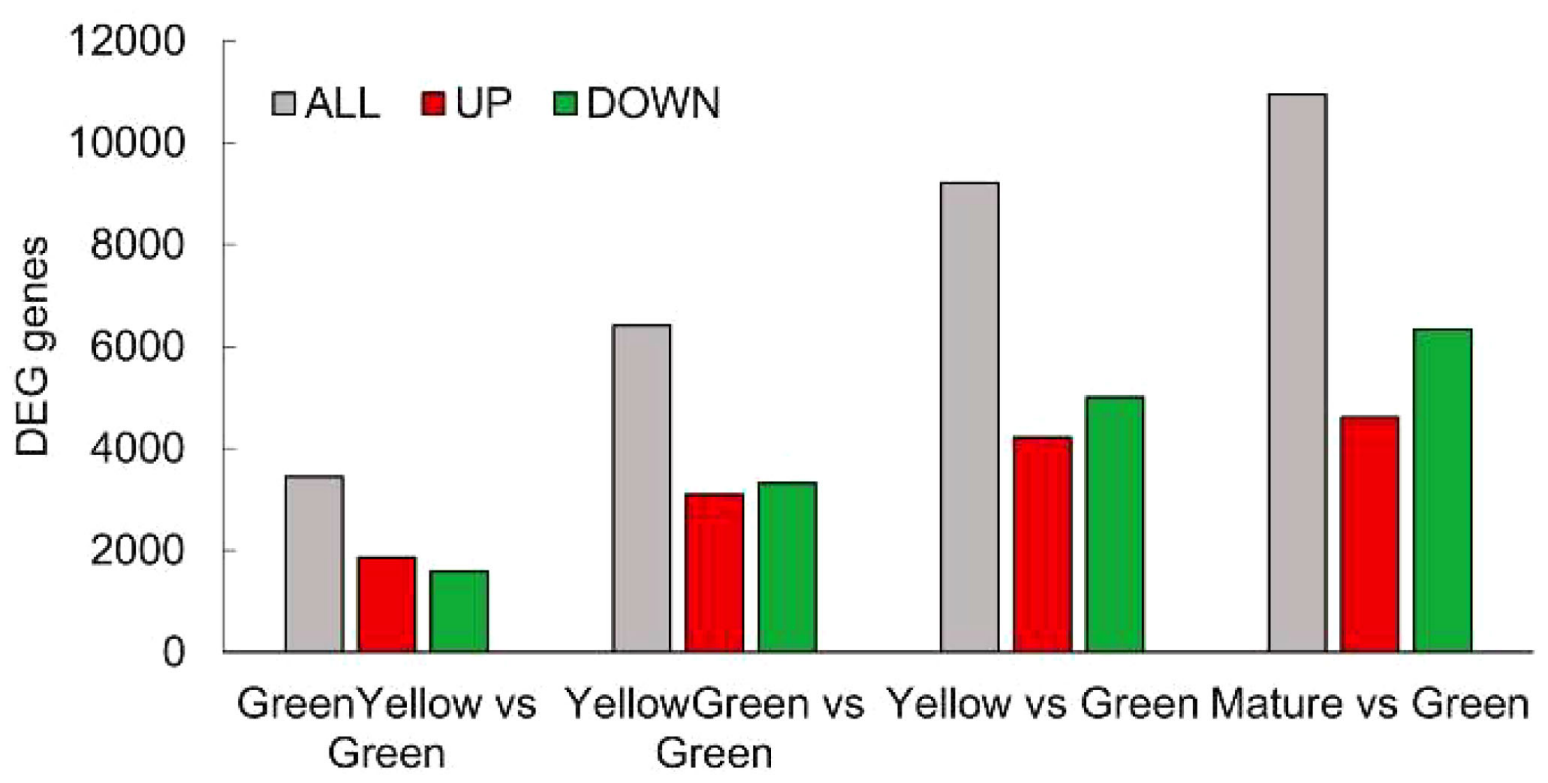
Distribution of DEG genes identified in the RNA-Seq analysis. Total (grey bars), up-regulated (red bars) and down-regulated (green bars) genes in each seed maturation stage. DEGs were identified by a log_2_ratio ≥ 1 and a padj ≤ 0.05.

To monitor the expression of genes encoding enzymes involved in seed oil biosynthesis during maturation, we first performed an RNA-Seq analysis to analyze transcriptome changes during maturation at the five stages described above. In general, and taking into account the latest annotation of the Pennycress genome available (García Navarrete et al., 2022), which estimated 28,034 genes from which 27,213 corresponded to protein coding genes, our RNA-Seq analysis identified 20,015 protein coding genes that covers a 73.54% of the total Pennycress genome. When pairwise comparisons were analyzed between seed maturation stages, the results indicated that 3,443 differentially expressed genes (DEGs) were identified in the GY vs G comparison, 6,406 in the YG vs G comparison, 9,214 in the Y vs G comparison, and 10,994 in the M vs G comparison (**Figure 2**). Interestingly, the number of identified DEGs increased during seed maturation and were not clustered to the initial maturation stages (**Figure 2**), indicating specific gene expression dynamics all-through the seed maturation process. The ratio of up-regulated to down-regulated genes also changed during seed maturation. While at the initial stages (G or GY) the up-regulated genes were slightly higher or similar to the down-regulated ones, at the late maturation stages (Y or M), the number of down-regulated genes was higher than that of the up-regulated genes (**Figure 2**).

#### *GO category and hierarchical* clustering analysis of DEGs

**Figure 3:**
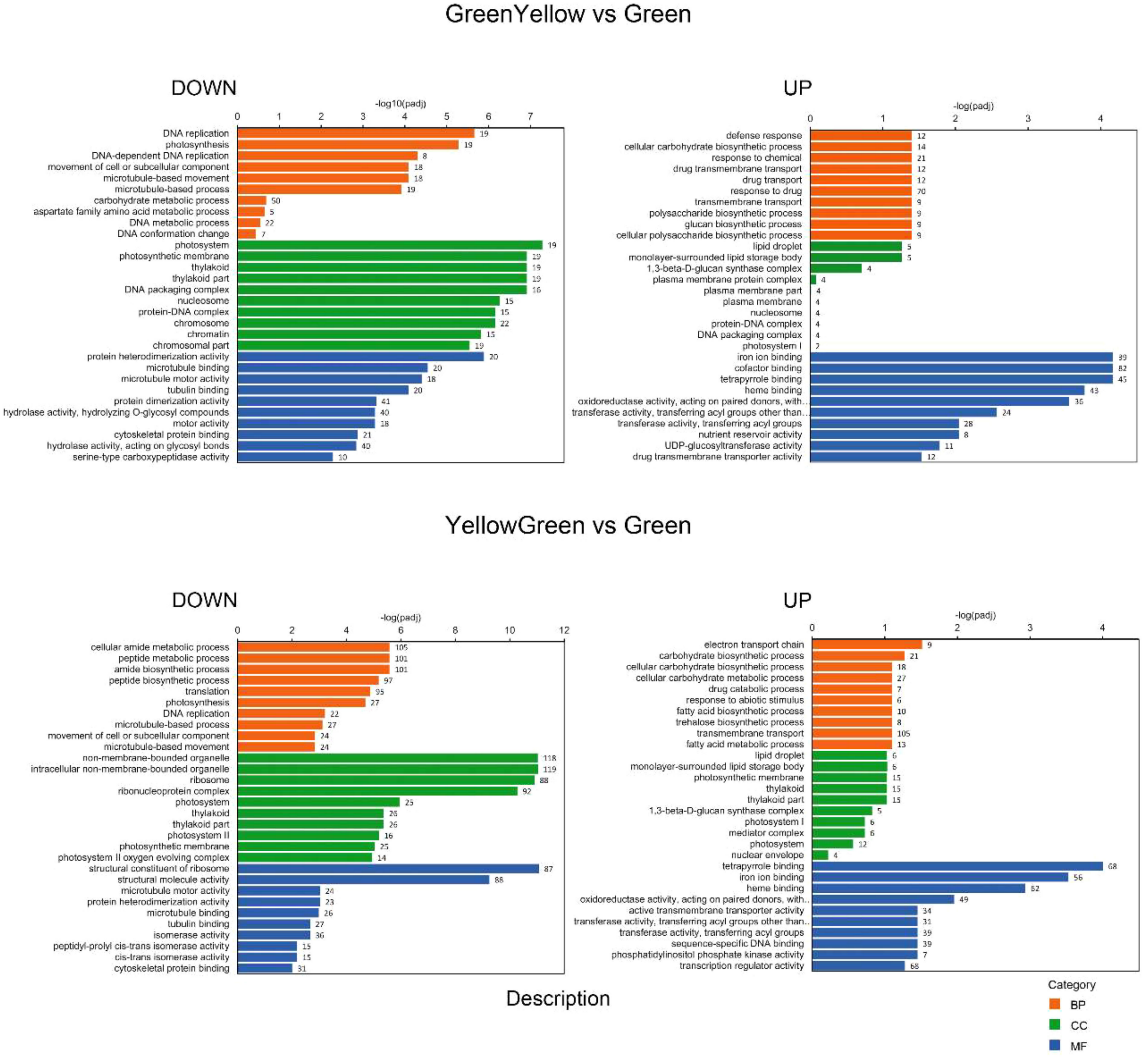
GO categorization analysis of DEG genes identified in the GREENYELLOW vs GREEN (upper panels) and YELLOWGREEN vs GREEN (lower panels) pairwise comparisons. Downregulated genes are shown on the left and upregulated ones on the right. Orange, green and blue colors indicate biological process (BP), cellular component (CC) and molecular functions (MF) categories respectively. Number of genes in each category is indicated in the bars.

**Figure 4:**
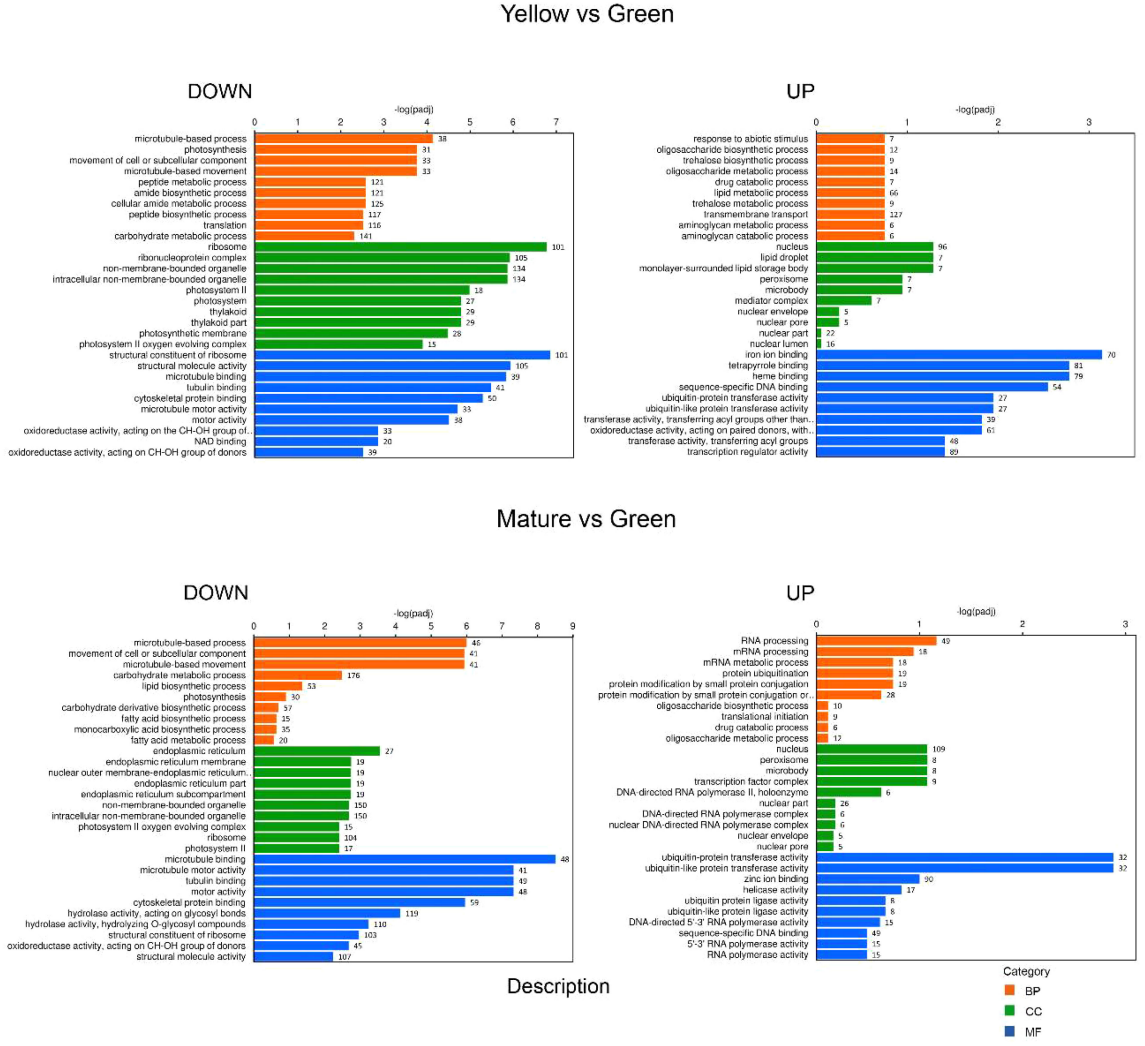
GO categorization analysis of DEG genes identified in the YELLOW vs GREEN (upper panels) and MATURE vs GREEN (lower panels) pairwise comparisons. Downregulated genes are shown on the left and upregulated ones on the right. Orange, green and blue colors indicate biological process (BP), cellular component (CC) and molecular functions (MF) categories respectively. Number of genes in each category is indicated in the bars.

Gene ontology (GO) analysis was performed with the identified DEGs for each maturation stage comparison. We split the results into down-regulated and up-regulated DEGs to facilitate their interpretation. With respect to biological process (BP), the GO categorization analysis of down-regulated DEGs showed a high proportion of genes involved in “DNA replication”, “DNA metabolic process” or “DNA conformational changes” and also in “photosynthesis”, at the GY, YG or Y when compared to the initial G stage (**Figures 3**, **4**). This association of DEGs found in BP was also confirmed in the cellular component (CC) category, where DEGs associated to “photosynthesis”, “thylakoid”, “chromosome”, and “nucleosome” or “DNA packaging” were found among the most represented associations. In the case of the up-regulated DEGs, at the BP category, DEGs associated to “fatty acid biosynthesis” and “fatty acid metabolism” were also highly represented in the YG and Y vs G comparisons (**Figures 3**, **4**). With respect to the CC category, genes associated with “lipid droplet” or “monolayer surrounded lipid storage”, as well as genes involved in “transferase activity” or “transfer of acyl groups” at the molecular function (MF) category, were highly represented in the GY, YG or Y vs G comparisons (**Figures 3**, **4**). Some relevant changes were observed when the GO analysis was performed on the M vs G comparison. Genes related with “lipid biosynthetic process” or “fatty acid biosynthetic process”, which were found in the up-regulated DEG list in the YG vs G or Y vs G comparisons, were found now at the down-regulated BP category in the M vs G comparison (**Figure 4**). This might be consistent with the fact that oil biosynthesis and oil filling might be reducing or even stopping at this seed maturation stage. Consistent with this, no genes related with lipid droplet formation were found in the GO analysis of the up-regulated DEGs (**Figure 4**). The same was true for DEGs with acyl group transferase activity in the MF category with respect to Y vs G or YG vs G (**Figure 4**). On the contrary, many upregulated DEGs related to RNA processing or protein ubiquitination were detected, consistent with the end of the seed maturation process.

**Figure 5:**
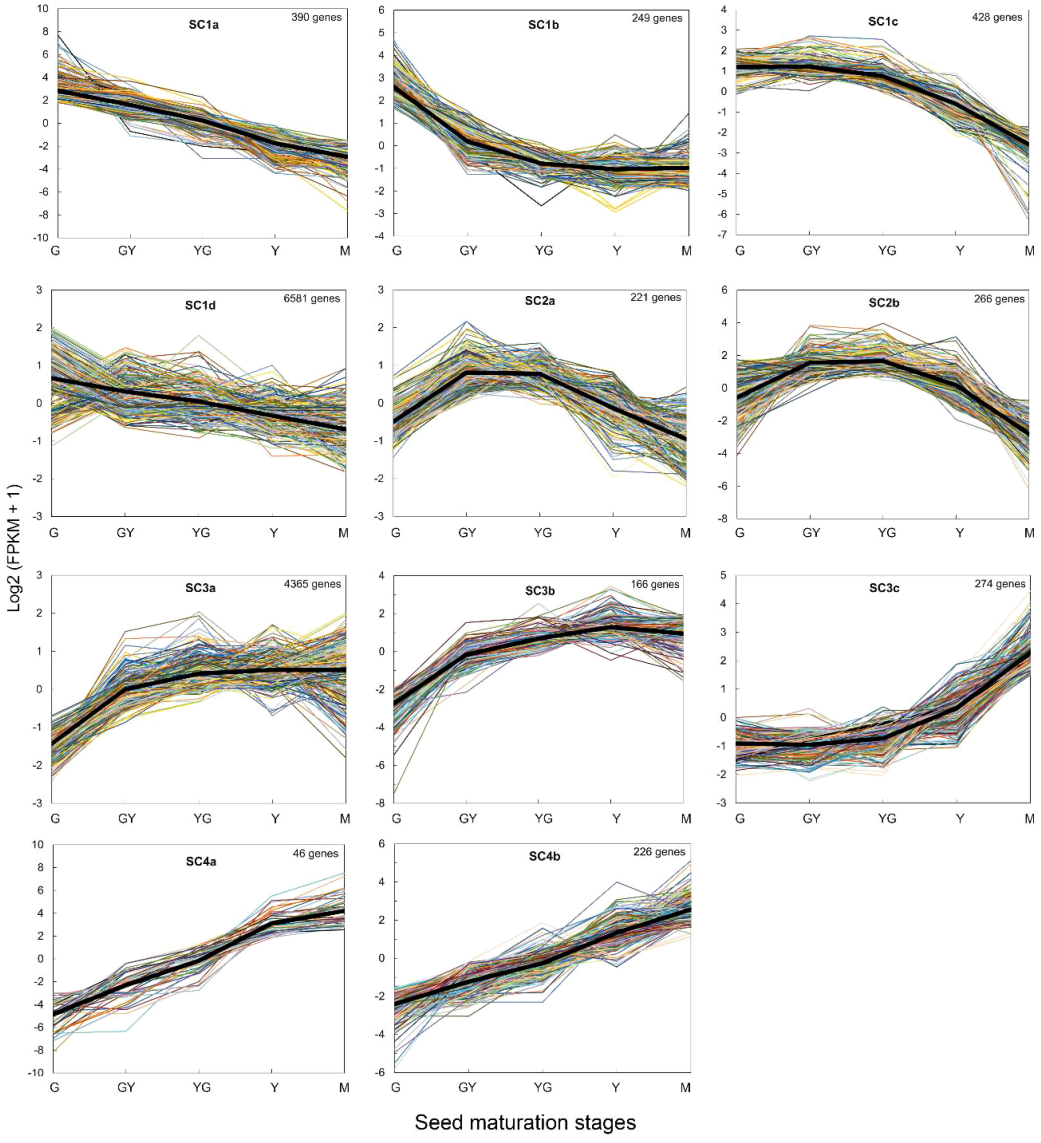
HCL clustering of genes obtained for each seed maturation stage in the RNA-Seq analysis. ClusterProfiler was used for the analysis. The black line in each cluster represents the average estimated variation for each cluster. The number of genes in each cluster is indicated in each figure.

We performed a gene clustering analysis of the DEGs using the log2 of FPKM values. The results showed that the gene expression patterns could be adjusted to minimally 4 main clusters that could be in some cases divided into sub-clusters. In general, the clustering analysis was consistent with the GO analysis. Cluster 1 (7,648 genes) grouped all DEGs that showed high expression levels at the G stage and then decreased in all the rest of the maturation stages to reach very low expression levels at the M stage (**Figure 5**). Genes encoding proteins involved in photosynthesis, photosystems, lipid transfer proteins (LTPs) or acyl carrier proteins (ACPs) which showed a strong decrease in their mRNA levels with seed maturation were detected in sub-cluster 1a (390 genes), (**Figure 5**). Sub-cluster 1d (6,581) grouped genes that showed more moderate decrease in their expression levels. Genes encoding LTPs, long acyl-CoA synthetases (*TaLACS1*, *TaLACS4* and *TaLACS9*), glycerol-3-phosphate acyltransferases (*TaGPAT1*, *TaGPAT6* and *TaGPAT7*), acyl carrier proteins (*TaACP1*, *TaACP2*, *TaACP4* and *TaACP5*) or genes encoding fatty acid desaturases like the endoplasmic reticulum (ER) omega-3 desaturase *TaFAD3* and the plastidial desaturases *TaFAD4* and *TaFAD6* were present in this sub-cluster (**Figure 5**).

Cluster 2 (487 genes) grouped all DEGs that increased from G to GY or YG stages and then decreased to Y and M final stages (**Figure 5**). Many seed storage proteins were detected in this cluster as well as other genes like the *TaFAE1* elongase, responsible of the synthesis of 22:1, some lipid transfer proteins (*TaLTP5*, *TaLTP20*), and also glycerol 3-phosphate acyltransferases like *GPAT4*. Cluster 3 (4,805 genes) grouped those DEGs which increased their expression from the initial stages of seed maturation and maintained their expression level to the end of the maturation of the seed (**Figure 5**). Sub-cluster 3a included most of the genes involved in TAG biosynthesis like *TaDGAT1*, *TaPDAT2*, *TaLPAT1*, two *GPATs* (*TaGPAT5* and *TaGPAT9*), and also genes encoding oleosins (*TaOLE1*, *TaOLE2*), OBAPs (*TaOBAP2B*) or Seipins (*TaSEIPIN2*), (**Figure 5**). Sub-cluster 3c (166 genes) grouped genes like *TaOBAP1A*, *TaOBAP2A*, *TaSEIPIN1*, all encoding proteins involved in lipid droplet accumulation (**Figure 5**). Finally, Cluster 4 (272 genes) grouped all the genes that increased their expression levels all through the seed maturation process, being higher at the mature stage (**Figure 5**). Many late embryogenesis abundant proteins like *TaLEA1* were detected in this cluster.

### Expression dynamics of genes involved in fatty acid biosynthesis and modification

**Figure 6:**
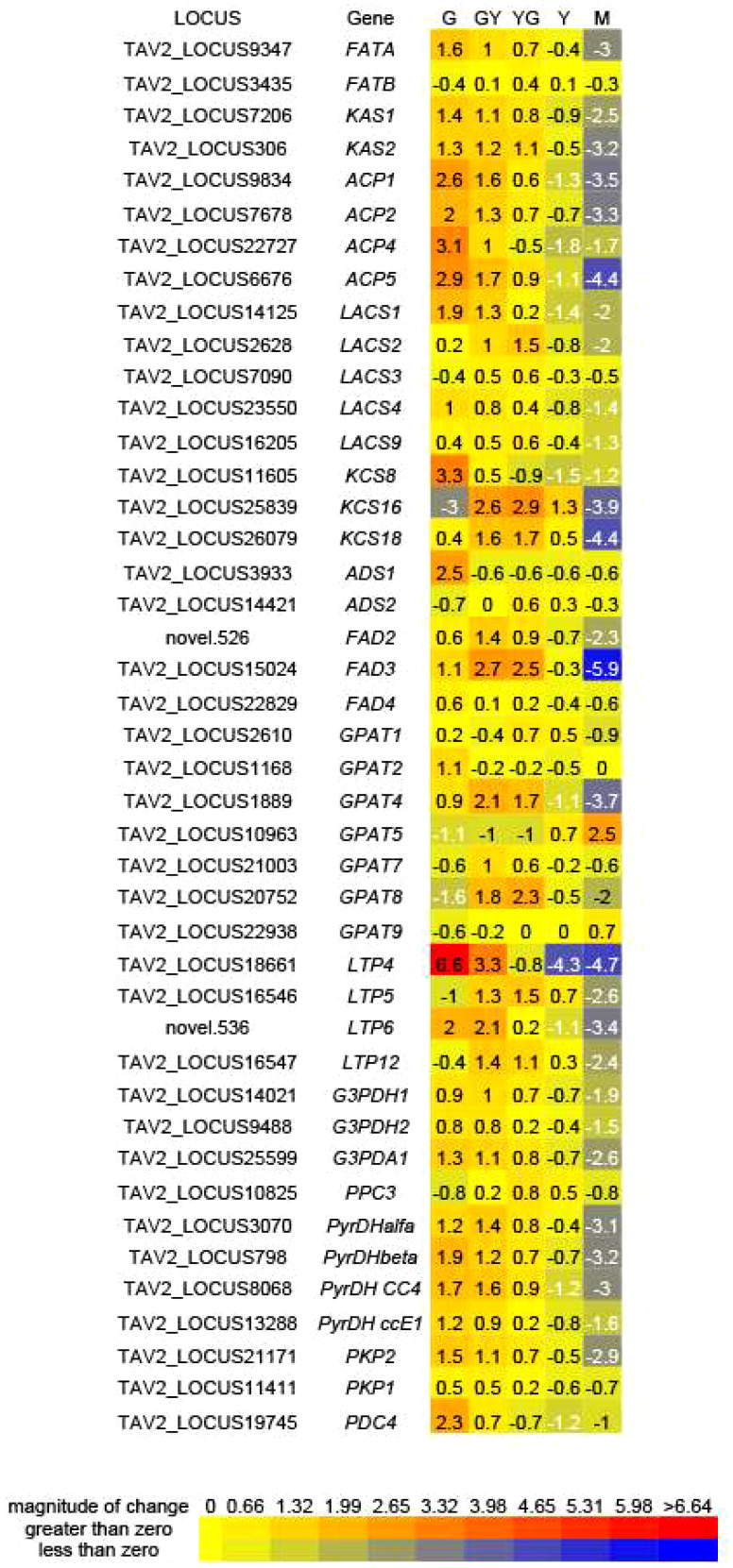
Differential expression of genes related to carbon assimilation and fatty acid biosynthesis for each of the five seed maturation stages used in this work. Values represent average log_2_(fpkm +1) values from each of the biological repeats and were used to generate heatmaps from https://bar.utoronto.ca/ntools/cgi-bin/ntools_heatmapper_plus.cgi. The *Thlaspi arvense* annotated genome (Nunn et al., 2022) was used for identification of the gene ID. FATA and FATB, fatty acid acyl ACP thioesterases; KAS, ketoacyl-ACP synthases; ACP, acyl carrier proteins; LACS, long-chain acyl-CoA synthetases; ADS, Acyl desaturase; FAD, fatty acid desaturase, GPAT, glycerol 3-phosphate acyltransferase: LTP, lipid transfer protein; G3PDH. Glycerol 3-phosphate dehydrogenase; PPC, phosphoenolpyruvate carboxylase; PyrDH, pyruvate dehydrogenase; PKP, phosphoenolpyruvate kinase; PDC, phosphoenolpyruvate decarboxylase.

Genes encoding enzymes involved in fatty acid biosynthesis or modification were analyzed into more detail. This included the two condensing enzymes, *TaKAS1* and *TaKAS2*, the two fatty acid ACP thioesterases, *TaFATA* and *TaFATB*, responsible of hydrolysing 16:0-ACP and 18:0-ACP substrates for export to the ER, acyl carrier proteins (ACPs) or Long Acyl Chain Synthetases (LACS). With the exception of *TaFATB*, that showed similar expression values, most, if not all these genes, showed higher expression values at the G, GY or YG stages and then decreased in the M stage (**Figure 6**). Similarly, several genes encoding 3-ketoacyl-CoA synthase family members, involved in the biosynthesis of VLCFAs, like *TaKCS8*, *TaKCS16* or *TaKCS18*, showed an expression pattern, higher at the early stages of maturation, similar to that of the FA biosynthetic genes (**Figure 6**). Expression of these genes, more concretely that of *TaKCS18 (FAE1*), was consistent with 20:1 and 22:1 fatty acid accumulation during Pennycress seed maturation (**Figure 1B**; Claver et al., 2017). Several enzymes involved in fatty acid modification like the Δ9 acyl-lipid desaturases *TaADS1* and *TaADS2*, involved in the desaturation of VLCFAs; the ER desaturases *TaFAD2* and *TaFAD3*, responsible of the biosynthesis of 18:2 and 18:3 fatty acids, or the plastidial *TaFAD4* desaturase, responsible of 16:1 synthesis in the plastid were also analyzed. *TaADS1* showed maximum expression at the G stage, decreasing with seed maturation, while *TaADS2* increased its expression from G to YG to decrease at the Y and M later maturation stages (**Figure 6**). The ER *TaFAD2* and *TaFAD3* desaturases also increased their expression from G to GY (*TaFAD2*) or to YG (*TaFAD3*) to decrease in the latter maturation stages (**Figure 6**). *TaFAD4* also showed maximum expression at the G stage decreasing with seed maturation (**Figure 6**).

The glycerol 3-phosphate acyltransferase (GPAT) is the enzyme that catalyzes the first step in TAG biosynthesis through the Kennedy pathway. GPAT is capable of transferring an acyl group to the *sn-1* position of glycerol 3-P to generate lysophosphatidic acid (LPA). Plant GPATs are a multigenic family with different sub-cellular localizations and roles in lipid biosynthesis. In Arabidopsis, *At*GPAT1 was located in the mitochondria playing a central role in the differentiation of the tapetum, male fertility and pollen development (Zheng et al., 2003). *At*GAPT4 and *At*GAPT8 are involved in extracellular lipid barriers (Yang et al., 2010), while AtGAPT5 was involved in suberin formation in seed coats (Men et al., 2017). *At*GAPT9 has been directly involved in the synthesis of storage lipids (Shockey et al., 2016). Our RNA-Seq analysis showed a complex pattern of regulation of the *TaGPAT* genes. Thus, *TaGPAT1* and *TaGPAT2* showed fluctuations of their expression without great changes in the different maturation stages (**Figure 6**). Interestingly, *TaGPAT4* and particularly, *TaGPAT8*, showed an increase of their expression values from G to the GY or YG stages to the decrease dramatically at the latter Y and M ones (**Figure 6**). On the contrary, *TaGPAT9* and particularly *TaGPAT5*, showed a specific and important increase of their mRNA levels at the Y and M stages (**Figure 6**). Genes involved in lipid transport, like *TaLTP4*, *TaLTP5*, *TaLTP6* and *TaLTP12*, also showed a similar higher expression at the earlier stages of seed maturation and then decreasing in the later ones (**Figure 6**). Particularly, *TaLTP4* and *TaLTP6* showed an important modification of their expression values suggesting a relevant role in the transport of acyl lipids in the seed.

Glycerol-3-phosphate and acetyl-CoA are the carbon sources necessary for fatty acid and TAG biosynthesis in the seed. Several genes involved in carbon assimilation like glycerol-3-phosphate dehydrogenases (G3PDHs), pyruvate dehydrogenases (PyrDHs), phosphoenolpyruvate carboxylases (PEPC) or phosphoenolpyruvate carboxylases:kinases (PEPCK) were monitored. All of them showed a similar expression pattern to those involved in fatty acid biosynthesis or modification, with higher expression values at the earlier stages of seed maturation (G, GY) to then decrease in the later ones (Y, M), (**Figure 6**).

### Expression dynamics of genes involved in TAG biosynthesis during Pennycress seed maturation

**Figure 7:**
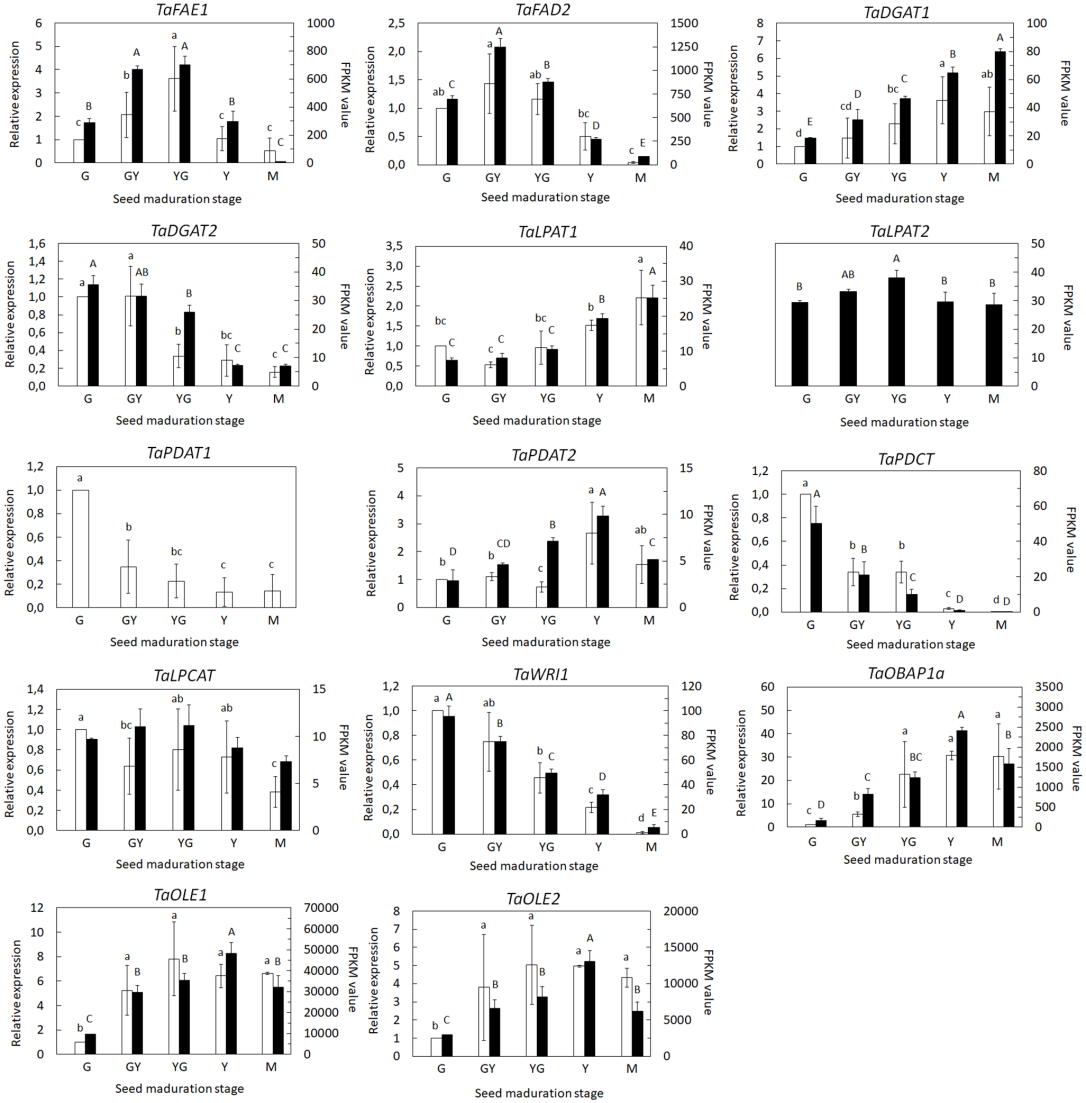
Expression profiling of individual genes and isoforms involved in VLCFA and TAG biosynthesis during seed maturation by qPCR (white bars) and RNA-Seq (black bars). For RNA-Seq data, expression levels are represented by FPKM values. Left y-axis represents qPCR relative expression data. Right y-axis represents FPKM values. The genes analyzed (*FAE1, FAD2, DGAT1, DGAT2, LPAT1, LPAT2, PDAT1, PDAT2, PDCT, LPCAT*, *WRI1, OLE1, OLE2 and OBAP1a*) are indicated in each figure. For qPCR analysis, data were obtained from three independent pools of seeds from five plants of each line. Data represent means ± SD of at least three biological replicates. Different lowercase letters and capital letters show significant differences among the different developmental stages during seed maturation of Pennycress (P < 0.05) for the RNA-Seq and qPCR data, respectively.

We focused our analysis on those genes involved in TAG biosynthesis as well as those involved in the biosynthesis and modification of fatty acids incorporated to TAG like erucic acid. On one hand, we used the RNA-Seq data (FPKM values) to monitor the expression of several selected genes. On the other hand, we performed a qPCR analysis on samples from each maturation stage of the same selected genes, comparing both expression data and validating the RNA-Seq results. Pennycress accumulates high levels of 22:1 during seed maturation (Claver et al., 2017; 2020). Expression of the *TaFAE1* gene, encoding the elongase responsible of 22:1 production, increased 3 to 5 fold between the initial G stage to the GY and YG maturation stages declining thereafter either in the RNA-Seq data and in the qPCR analysis (**Figure 7**). This induction of *TaFAE1* at the initial stages of Pennycress seed maturation could be consistent with the rapid availability of 22:1-CoA in the acyl-CoA pool for its early incorporation to total lipids as shown in **Figure 1B** and reported previously by our group in Pennycress accessions of European origin (Claver et al., 2017). A very similar gene expression profile was obtained for the *TaFAD2* desaturase, with a two-fold increase of mRNA levels obtained both at the RNA-Seq data and qPCR analysis at the GY stage to then decrease its mRNA levels upon Pennycress seed maturation (**Figure 7**). This again was consistent with the high 18:2 levels in total lipids at the early stages of seed maturation (**Figure 1B**).

*TaDGAT1* and *TaDGAT2* genes encode two diacylglycerol acyltransferases with high homology, 90.6 and 81.3% with respect to the Arabidopsis *AtDGAT1* and *AtDGAT2* genes, respectively (Routaboul et al., 1999; Shockey et al., 2006). *TaDGAT1* expression levels increased gradually during Pennycress seed maturation, particularly between the YG and Y stages, showing a 2,5-3 fold maximum increase at the Y stage (**Figure 7**). On the contrary, *TaDGAT2* expression results from both RNA-Seq and qPCR data showed higher mRNA levels at the initial maturation stages, G and GY, further declining upon seed maturation (**Figure 7**). It is worth mentioning that the FPKM values of both *TaDGAT1* and *TaDGAT2* genes indicated that *TaDGAT2* mRNA was more abundant than that of *TaDGAT1* at the G stage and similar at the GY one, suggesting a specific role of *TaDGAT2* in TAG biosynthesis, particularly at the initial stages of Pennycress seed maturation.

In higher plants, lysophosphatidic acid-acyltransferases (LPATs) are a multigenic family involved in DAG biosynthesis in the Kennedy pathway (Kim and Huang, 2004; Kim et al., 2005). Two *LPAT* genes, *TaLPAT1* and *TaLPAT2*, with high homology to their Arabidopsis orthologues, were detected in the Pennycress genome. *TaLPAT1* expression followed a similar pattern to that of *TaDGAT1*, with a mRNA increase from the YG stage to the Y and M stages between 2-3.5 fold, both in the RNA-Seq and qPCR data (**Figure 7**). On the contrary, *TaLPAT2* showed very subtle changes in its expression.

The expression of genes encoding enzymes of the acyl-editing pathway was also monitored. As occurred with the two *DGAT* genes, two *TaPDAT* genes, encoding the phospholipid-diacylglycerol acyltransferases responsible of the biosynthesis of TAG through the acyl-editing pathway (Zhang et al., 2009) were detected in the Pennycress genome with high homology, 87 and 88% with respect to the Arabidopsis *AtPDAT1* and *AtPDAT2* genes, respectively. Both *PDAT* genes showed completely different expression patterns. It is worth mentioning that the *TaPDAT1* gene was not detected in the RNA-Seq data and only the *TaPDAT2* gene was found. qPCR data indicated that *TaPDAT1* mRNA levels were high at the initial stages of seed development and then rapidly declined (**Figure 7**). On the contrary, the *TaPDAT2* gene showed a continuous increase in mRNA levels upon seed maturation reaching maximum expression levels at the Y stage both in the qPCR and in RNA-Seq data. These results might suggest different roles for both PDAT enzymes at the early (*PDAT1*) or late (*PDAT2*) stages of seed maturation. The other enzyme of the acyl editing pathway, *TaLPCAT*, responsible of the reincorporation of an acyl group to PC, maintained its expression levels between the G to the YG stage to then slowly decreased at the Y and M stages, suggesting that *LPCAT* expression and/or activity might not be limiting for seed oil accumulation. Interestingly, the expression of the *TaPDCT* gene, involved in PC-derived DAG interconversion, showed a similar expression pattern to that of *TaPDAT1*, with high mRNA accumulation at the early stages of seed maturation (**Figure 7**).

Expression of *WRINKLED1*, the TF involved in the control of many genes of the lipid biosynthetic pathway in the seed (Cernac and Benning, 2004; Baud et al., 2007), showed higher expression at the early stages of seed maturation, consistent with the upregulation of seed oil biosynthetic genes (**Figure 7**).

**Figure 8:**
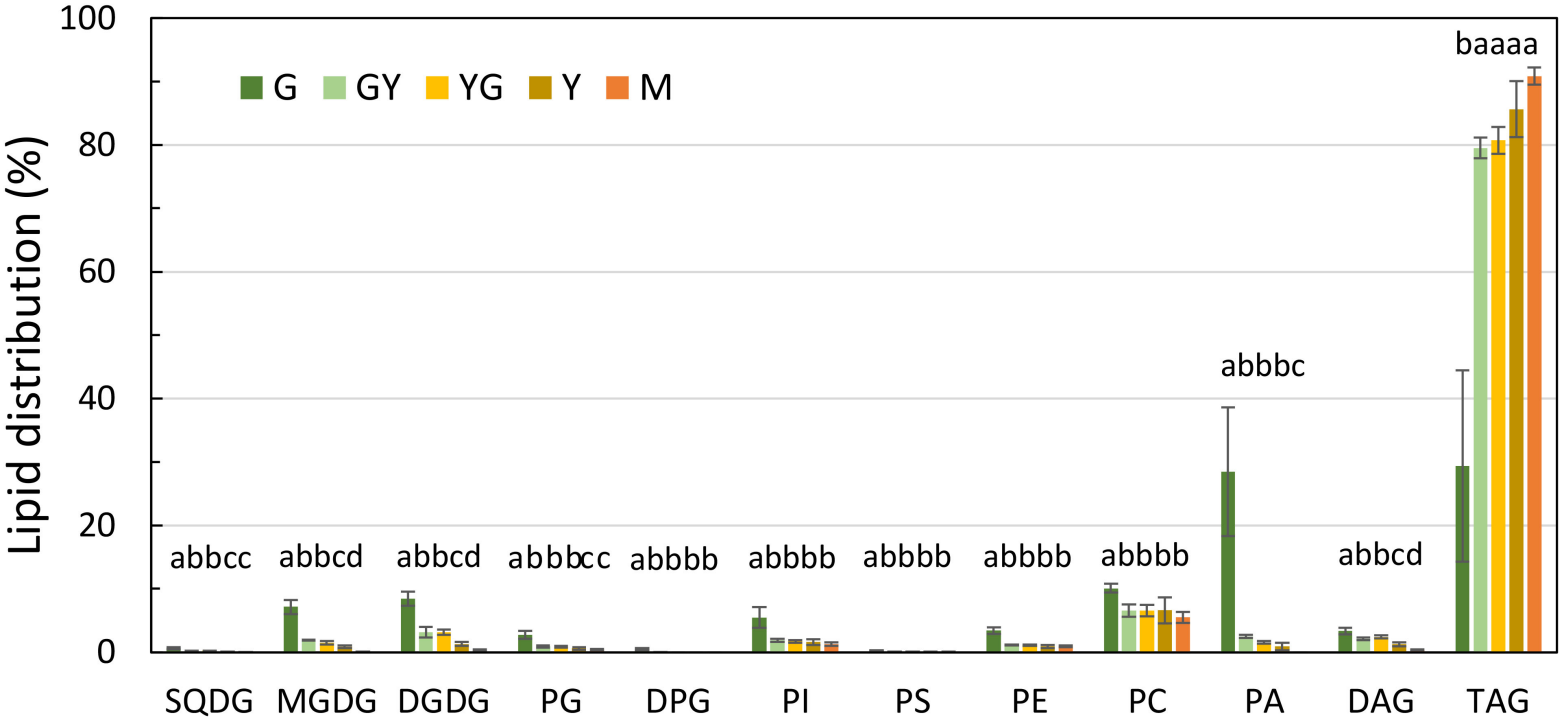
Glycerolipid distribution during Pennycress seed maturation. Values expressed in percentage of total lipids. Seed maturation stages are indicated in the figure. Values presented are average of three determinations from two biological replicates and error bars represent SE. For the same lipid class, different letters indicate significant differences among seed maturation stage at *P <*0.05. DAG, diacylglycerol; DGDG, digalactosyldiacylglycerol; DPG, diphosphatidylglycerol; MGDG, monogalactosyldiacylglycerol; PA, phosphatidic acid; PC, phosphatidylcholine; PE, phosphatidylethanolamine; PG, phosphatidylglycine; PI, phosphatidylinositol; PS, phosphatidylserine; SQDG, sulfoquinovosyldiacylglycerol; TAG, triacylglycerol.

Finally, we monitored the expression of genes involved in lipid droplet formation. These lipid droplets increase their number and size upon seed maturation (Farese and Walther, 2009). Genes encoding two oleosins, *TaOLE1* and *TaOLE2*, showed a similar increase in mRNA levels both in the RNA-Seq data and in the qPCR analysis, with maximum values between the YG and Y stages (**Figure 7**), consistent with higher TAG accumulation (**Figure 8**). Interestingly, *TaOBAP1a* showed a similar increase during seed maturation although the extent of these changes seemed to be much higher than Oleosins at least at the transcript levels (15-30 fold at the Y stage; **Figure 7**).

### Glycerolipid analysis during Pennycress seed maturation

Glycerolipid analysis of the different lipid species present in the Pennycress seeds was carried out by LC-MS (Jouhet et al., 2017). This analysis showed some significant changes in some lipid classes during Pennycress seed maturation. Thus, at the initial G stage, several lipid classes showed different relative abundances like TAG (29.3%), PA (28.5%), PE (3.4%) or the plastidial lipids MGDG (7.1%) or DGDG (8.4%), (**Figure 8**). Other lipid classes like PG (2.7%), PC (10.1%) or DAG (3.3%) were also detected in this initial maturation stage (**Figure 8**). Upon seed maturation, TAG levels showed the highest increases among the different lipid classes, with values ranging from 79.5% at the GY stage to 85.6% at the Y stage or 90.8% at the M stage (**Figure 8**). Conversely, other lipid species like MGDG or DGDG showed a decrease during seed maturation, decreasing to 0.8% at the Y stage or to 0.09% at the M stage for MGDG (**Figure 8**). Other lipid classes like PC or DAG did not show such relevant changes. Thus, PC levels decreased during Pennycress seed maturation, with relative levels ranging from 10.1% at the G stage to 5.4% at the M stage (**Figure 8**). Similarly, DAG levels kept close to the 3% range at the G, GY, and YG stages, showing a decrease to lower values at the late M stages (0.8%), (**Figure 8**). It is worth mentioning that PC levels were always higher than those from DAG in all seed maturation stages.

**Figure 9:**
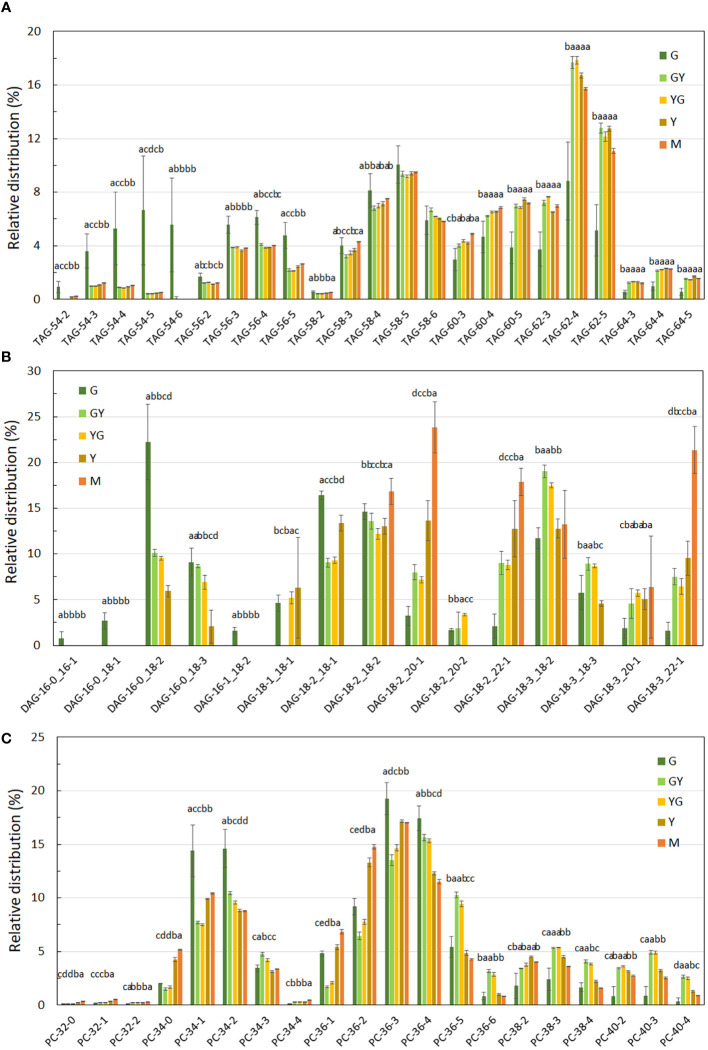
Fatty acid distribution in TAG **(A)**, DAG **(B)** and PC **(C)** lipid fractions during Pennycress seed maturation. Values expressed in percentage of total lipids for each class. Seed maturation stages are indicated in the figure. Values presented are average of three determinations from two biological replicates; error bars represent SD. DAG, diacylglycerol; PC, phosphatidylcholine; TAG, triacylglycerol. Different letters above the bars indicate significant differences among the different seed maturation stages for each species (P< 0.05).

Quantitative analysis of the acyl composition of the different lipid classes revealed changes in their distribution with Pennycress seed maturation. **Figure 9A** shows the acyl composition of TAG, the major lipid fraction as well as those from DAG and PC as intermediate species during TAG biosynthesis. Fatty acid distribution in TAG showed that at the G stage, 58:4 (18:1/18:2/22:1) and 58:5 (18:2/18:2/22:1) together with 62:4 (22:1/18:2/22:1) were the most abundant TAG species detected in the LC-MS analysis (**Figure 9A**). Other TAG species like 56:3 (16:0/18:2/22:1), 54:4 (18:1/18:1/18:2) or 54:5 (18:1/18:2/18:2) were also very abundant at this initial G stage (**Figure 9A**). TAG 64:4 (22:1/18:2/24:1), as well as 64:3 or 64:5, was also present at this initial maturation stage although in very low amounts (less than 1%), (**Figure 9A**). It is worth mentioning that although TAG species containing 16 and 18 carbon fatty acids were highly abundant at this initial stage, many of them already contained 22:1, indicating a rapid incorporation of 22:1 to TAG even at the younger stages.

TAG species containing VLCFAs, particularly 22:1, increased upon seed maturation. Thus, 62:4 levels doubled from the G to the GY or YG stages (**Figure 9A**). The same occurred with 62:5 (18:3/22:1/22:1), being now 62:4 and 62:5 the most abundant TAG species in the rest of the stages upon Pennycress seed maturation. Similarly, TAG species containing 22:1 and 24:1 like 64:4 or 64:5, although much less abundant than 62:4 or 62:5, also increased with seed maturation (**Figure 9A**). Levels of 58:4 and 58:5 remained similar at the rest of the maturation stages. On the contrary, levels of TAG species like 54:4 or 54:5, containing 16:0, 18:1 and 18:2 fatty acids, that were very abundant at the G stage decreased rapidly with seed maturation (**Figure 9A**). The fatty acid composition of DAG was also analyzed. 34:2 (16:0/18:2) was the major DAG species detected at the initial G stage (**Figure 9B**), representing a 22.21% of the total DAG detected in this stage. Other abundant DAG species were 36:3 (18:1/18:2), 36:4 (18:2/18:2), 36:5 (18:2/18:3) or 36:6 (18:3/18:3), (**Figure 9B**). DAG species containing VLCFAs like 38:4 (18:3/20:1), 40:3 (18:2/22:1) or 40:4 (18:3/22:1) were also present at the G stage although their relative abundance was lower than those species containing C16 or C18 fatty acids (**Figure 9B**). This distribution changed dramatically upon seed maturation. Thus, 40:3 (18:2/22:1) levels increased up to 3-fold from 2.6% to 9.0 and 8,8% at the GY and YG stages, respectively, when compared to the G stage, reaching even higher 12.7% and 17.8% relative levels at the Y and mature M stages, respectively, (**Figure 9B**). DAG 38:3 (18:2/20:1) and 40:4 (18:3/22:1) also increased dramatically with Pennycress seed maturation with values of 3.2% at the G stage to 8.0% (GY), 7.2% (YG), 13.6% (Y) and 23.8% (M) in the case of 38:3 or 7.5%(GY), 6.4% (YG), 9.5% (Y) and 21.3% (M) for 40:4, indicating a higher accumulation of VLCFAs, particularly 22:1 in DAG during Pennycress seed maturation. In fact, 38:3, 40:3 and 40:4 were the major DAG species in mature seeds (**Figure 9B**). This increase in VLCFAs containing species was concomitant with the decrease of DAG 34:2, which was the most abundant one at the G stage (**Figure 9B**). Other DAG species like 36:4, remained almost constant with little variations at the different seed maturation stages (**Figure 9B**).

Acyl group distribution in phospholipids was also analyzed. 34:1 (16:0/18:1), 34:2 (16:0/18:2), 36:3 (18:1/18:2) and 36:4 (18:2/18:2) were the most abundant PC species detected in the initial G stage, with relative amounts of 14.4%, 14.6%, 19.3% and 17.4%, respectively (**Figure 9C**). PC species containing 20:1 like 38:2 (18:1/20:1), 38:3 (18:2/20:1), or 38:4 (18:3/20:1) were also detected although in much lower amounts (1.6-2.4%) when compared to PC species containing C16 and C18 fatty acids (**Figure 9C**). PC species containing 22:1 like 40:2, 40:3 or 40:4 were also detected in the G initial stage although in very low amounts (0.8%; **Figure 9C**). Upon seed maturation, PC species like 34:1, 36:3 and 36:4 showed a decrease in their relative levels while other like 36:5 (18:2/18:3) or 36:2 (18:1/18:1) increased their levels with seed maturation. Interestingly, PC species containing VLCFAs increased their relative abundance with seed maturation. Thus, PC species like 38:2, 38:3, 38:4 (containing 20:1) and 40:2, 40:3 and 40:4 (containing 22.1) increased their levels, particularly from the G to the GY and YG stages (**Figure 9C**). It is worth mentioning that our data of the presence and distribution changes upon seed maturation of PC species containing VLCFAs differed from those previously reported by Romsdahl et al. (2022) that detected PC species containing VLCFAs at very low levels (if any) in their analysis. Acyl group distribution in other phospholipids showed a similar fatty acid composition with respect to that from PC, with species containing C16 and C18 fatty acids as major species, presence of VLCFAs in lower amounts at the initial stages of seed maturation that increases upon seed maturation (**Supplementary Figure S3**). Particularly interesting were the results obtained with PS that showed a high accumulation of 40:3 (18:2/22:1) and 40:4 (18:3/22:1) species upon seed maturation.

### HPTLC-ESI-MS characterization and positional analysis of TAG species by tandem mass spectrometry

We decided to monitor how the erucic acid, as well as other acyl groups, were incorporated to TAG, with particular interest in the positional analysis of the different acyl groups esterified to TAG. To that end, we used a lipidomics approach based in the application of HPTLC - UV densitometry - MS to analyze the chemical composition of the different TAG species at each seed developmental stage and then couple this analysis with tandem mass spectrometry to study the fragmentation pattern of these TAG species and obtain positional information. HPTLC-ESI-MS has proven to be useful for lipidomic analysis in complex lipid mixtures (Jarne et al., 2018, 2021; Sancho-Albero et al., 2022). This analysis focused on TAGs as it constitutes the major fraction of the total seed lipids in Pennycress (**Figure 8**; Claver et al., 2017). Two different lipid extract samples per stage (G, GY, YG, Y and M), which corresponded to two different extraction batches, were analyzed by HPTLC-densitometry-MS. Percentages of TAGs in samples, as well as intra- and inter-plate HPTLC repeatability results (expressed in Area counts) for the separated TAG peaks are presented in **Supplementary Table S4**, including the average, the relative standard deviation (RSD%), and the coefficient of variation for a confidence interval of 95% (CV).

### Identification of TAG species by HPTLC-Ion Trap MS

**Figure 10:**
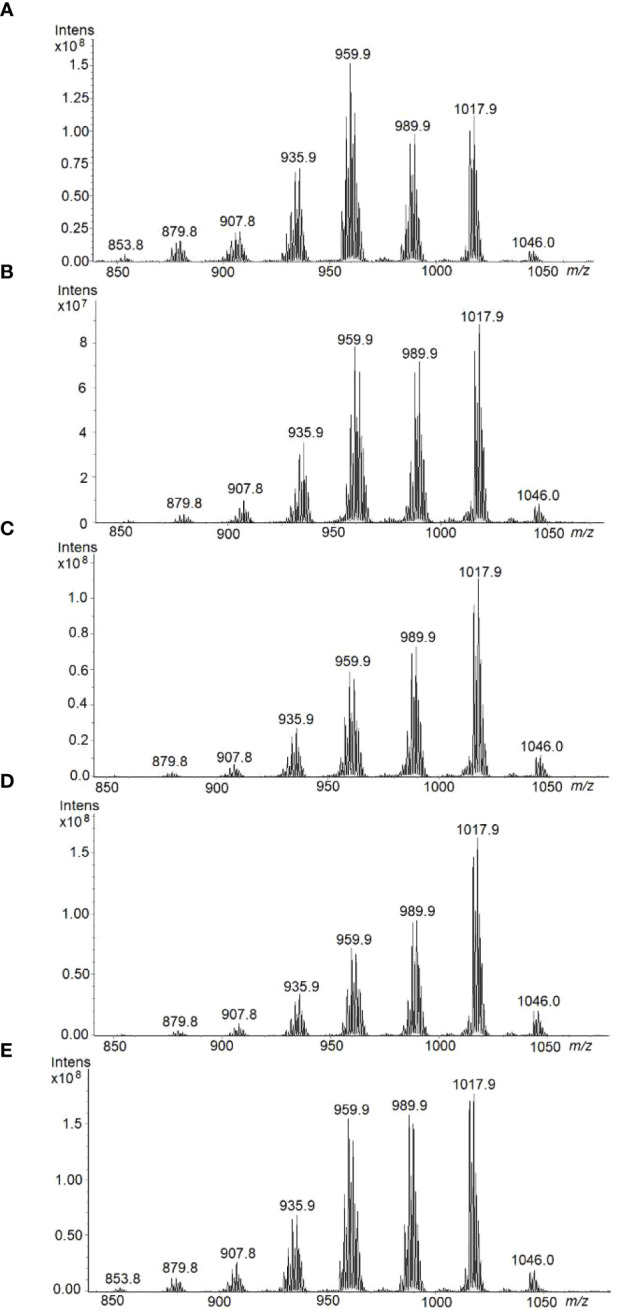
HPTLC-ESI^+^-MS profiles of TAG fraction separated by HPTLC and extracted from the plate, using the interface, for each of the different Pennycress seed maturation stage: **(A)** GREEN, **(B)** GREEN-YELLOW, **(C)** YELLOW-GREEN, **(D)** YELLOW, and **(E)** MATURE.

**Figure 10** shows the HPTLC-ESI^+^-MS spectra of TAG zones corresponding to each Pennycress seed maturation stage. For all samples, mass spectra were recorded at the same ionization time and conditions, in order to compare relative ion intensities of different TAG species in each maturation stage. In general, eight major TAG species were identified in all seed maturation stages with maxima intensities at *m/z* 853.8; 879.8; 907.8; 935.9; 959.9; 989.9; 1017.9 and 1046.0, (**Figure 10**). These molecular species corresponded to 54:3, 56:3, 58:5, 60:4, 62:4 and 64:4, respectively. Although a quantitative analysis is excluded, there is an ion intensity-concentration relationship for each sample that allows the comparison between ion intensities from the ESI^+^-MS spectra of each sample. Accordingly, at the early G and GY stages, TAG species with *m/z* 959.9 (58:5) was the major TAG species (**Figure 10**). Other TAG species identified in the analysis were 56:3 (*m/z* 935.9), 60:4 (*m/z* 989.9) and 62:4 (*m/z* 1017.9), (**Figure 10**). This distribution was similar to that obtained by LC-MS (**Figure 9A**). Upon seed maturation, the distribution of TAG species at the YG and Y stages showed a change with respect to the G and GY initial stage: an increase of TAG 60:4 (*m/z* 989.9) and 62:4 (*m/z* 1017.9) corresponding to the species containing 20:1/18:2/22:1 and 22:1/18:2/22:1, respectively was observed (**Figure 10**). Finally, at mature stage (M), TAG species like 58:5 (*m/z* 959.9), 60:4 (*m/z* 989.9) and 62:4 (*m/z* 1017.9) were the most abundant ones (**Figure 10**). 24:1 in the TAG 64:4 (ion at 1046.0 *m/z*) species was also detected in the analysis. Results from HPTLC-MS using an ion trap were in good agreement with those from LC-MS using a triple quadrupole (**Figure 9A**) indicating that, upon maturation, an increase in TAG species containing VLCFAs like 22:1 or 20:1 occurred. These results validate the use of HPTLC-ESI-MS technology for the analysis of TAG species in complex lipid mixtures like Pennycress seed lipid fractions.

### *sn*-positional analysis

TAGs are weakly basic esters and, under the HPTLC and ESI^+^ conditions readily lead to the formation of [TAG+Na]^+^, which in our case can be fragmented to yield structural information (Jarne et al., 2018, 2021; Sancho-Albero et al., 2022). According to Ramaley et al. (2015), [M+Na]^+^ adducts from ion trap are the most suitable for TAG regioisomer analysis as they produced the most consistent level of positional sensitivity for the fragmentation. The preferential loss of the fatty acid at positions *sn*-1/3 seems to be general for TAG molecules regardless of energy and instrumentation employed, leading to the formation of two ions of similar abundance corresponding to the losses of the fatty acids substituents at *sn*-1 and at *sn*-3 and those are significantly more abundant than the ion corresponding to the loss of the fatty acid substituent at *sn*-2 (Hsu and Turk, 2010). Thus, the intensities of the resulting fragment ions reflect the FA distribution in the glycerol backbone.

**Figure 11:**
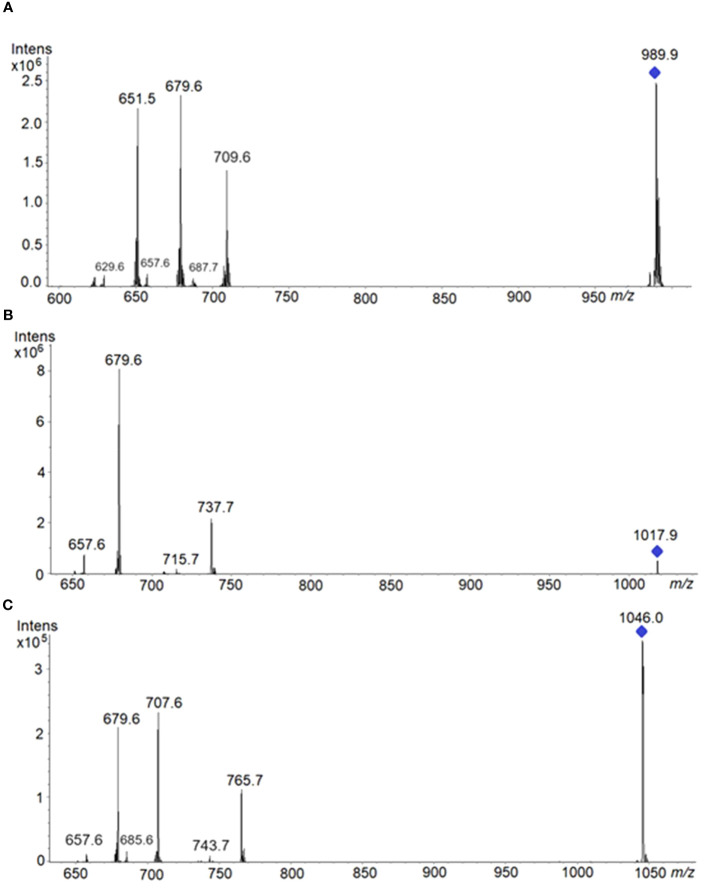
HPTLC-ESI^+^-MS/MS spectra of the following precursor ions: **(A)** at 989.9 *m/z*, spectrum showed three ion products corresponding to losses of fatty acyl substituents as fatty acids: at 679.6 *m/z* (most intense) which corresponds to [M+Na−R_1,3_COOH]^+^, R_1,3_=C(20:1)FA; at 651.5 *m/z* which corresponds to [M+Na−R_3,1_COOH]^+^, R_3,1_= C(22:1)FA; at 709.6 *m/z* which corresponds to [M+Na−R_2_COOH]^+^, R_2_= C(18:2)FA. Product ions at low intensities: 657.6 *m/z* which corresponds to [M+Na−R_1,3_COONa]^+^; 629.6 *m/z* which corresponds to [M+Na−R_3,1_COONa]^+^; and 687.7 *m/z* which corresponds to [M+Na−R_2_COONa]^+^. **(B)** at 1017.9 *m/z*. Product ions are explained in the text. **(C)** at 1046.0 *m/z*, spectrum showed three ion products corresponding to losses of fatty acyl substituents as fatty acids: at 707.6 *m/z* (most intense) which corresponds to: [M+Na−R_1,3_COOH]^+^, R_1,3_= C(22:1)FA; and at 679.6 *m/z* which corresponds to [M+Na−R_3,1_COOH]^+^, R_3,1_= C(24:1)FA; and 765.7 m/z which corresponds to [M+Na−R_2_COOH]^+^, R_2_= C(18:2)FA. Product ions at low intensities: 657.6 *m/z* corresponds to [M+Na−R_3,1_COONa]^+^; 685.6 *m/z*, to [M+Na−R_1,3_COONa]^+^; and 743.7 *m/z*, to [M+Na−R_2_COONa]^+^.

TAG regioisomers can be identified with MS^n^ methods, but analysis of TAG enantiomers is not possible because the fragmentation methods cannot distinguish between *sn*-1 and *sn*-3 fatty acids due to the identical fragmentation efficiencies of fatty acids from these positions. In natural products, many isobaric TAG species, including isomers may produce shared isobaric fragment ions, making the analysis of TAG regioisomers even more challenging. However, on several occasions where the most abundant ion comes from a single triad of fatty acid composition, we have been able to identify the fatty acid in the *sn*-2 position. Ions *m/z* corresponding to TAG species are reported in **Table 1** along with their fragmentation patterns (MS^2^). Therefore, ion at m/z 1017.9 corresponds to the sodium adduct of TAG (62:4) [C_65_H_118_O_6_Na]^+^. As the stability of [M+Na]^+^ was high, a consecutive fragmentation was achieved in the ion-trap MS to have verification of identity. Hence, the respective HPTLC-ESI^+^-MS/MS spectrum of the precursor ion at *m/z* 1017.9 showed two ion products corresponding to losses of fatty acyl substituents as fatty acids: at *m/z* 679.6 (most intense) which corresponds to [M+Na−R_1,3_COOH]^+^, R_1,3_= C(22:1) fatty acids, and at *m/z* 737.62 which corresponds to [M+Na−R_2_COOH]^+^, R_2_= C(18:2) fatty acids; and two much less abundant ions products corresponding to losses of fatty acyl substituents as their sodium salts: 657.6 *m/z* which corresponds to [M+Na−R_1,3_COONa]^+^, R_1,3_ = C(22:1) fatty acids, and 715.7 *m/z* which corresponds to [M+Na−R_2_COONa]^+^, R_2_= C(18:2) fatty acid (**Table 1**, **Figure 11**). ESI MS/MS spectra were carried out using He as the collision gas, an optimal amplitude voltage of 0.6 V and an isolation width for the precursor ion of 1 *m/z* units (**Table 1**, **Figure 11**). Results are consistent with a TAG structure of 22:1/18:2/22:1 with linoleic acid at the *sn*-2 position. This TAG is already present at the youngest state, in all stages, and becoming the most important in GY, YG, Y and M. It was also possible to identify *sn*-2 position in the ion at *m/z* 989.8 and in the ion at *m/z* 1046.0. In the other ions at *m/z* 907,8, 935,9 and 959, 9, although fragments compatible with 18:2 at *sn-2* were obtained, it was more difficult to identify the *sn-*2 position since several TAG species can contribute to the same ion. In these cases, this analysis should not be used as the basis for excluding the presence of other isomers.

**Table 1:** TAG molecular species corresponding to a unique combination of fatty acyls.

Exact mass	[TAG+Na]^+^ *m/z*	[M+Na−RCOOH]^+^		[M+Na−RCOONa]^+^	Molecular Formulae	Unique TAG
		***m/z***	**- (Cx;y)FA**	***m/z***		
989.8508	989.9	651.5 679.6 709.6	- C22:1 - C20:1 -C18:2 (*sn*-2)	629.6 657.6 687.7	C_63_H_114_NaO_6_ TAG 60:4	20:1/18:2/22:1
1017.8821	1017.9	679.6 737.7	- C22:1 - C18:2 (*sn*-2)	657.6 715.7	C_65_H_118_NaO_6_ TAG 62:4	22:1/18:2/22:1
n.m.	1046.0	679.6 707.6 765.7	- C24:1 - C22:1 - C18:2 (*sn*-2)	657.6 685.6 743.7	C_67_H_122_NaO_6_ TAG 64:5	22:1/18:2/24:1

Precursor and product ions (m/z, MS^2^) from TAG peaks separated and identified using HPTLC-ESI^+^-MS from total lipid extracts of Pennycress seeds. Exact mass by HR-MS. Isolation window (MS^2^, ion trap): ± 0.5 u.m.a.

## Discussion

In this work, we have studied seed oil biosynthesis in the biofuel feedstock Pennycress. Our goal was to analyze the pathways involved in TAG biosynthesis in this species, determining how erucic acid was incorporated to TAG and the contribution of the different TAG biosynthesis pathways during Pennycress seed maturation. This question was addressed through a transcriptomic together with a lipidomic approach to analyze the expression pattern of genes involved in fatty acid and TAG biosynthetis during seed maturation and to correlate these results with changes in glycerolipid and acyl group distribution. Further information of the incorporation of VLCFAs to TAG was obtained through positional analysis. This knowledge will help us to understand the molecular and biochemical determinants of the different seed oil content and fatty acid composition of the Pennycress seed oil when compared to other Brassicaceae like Arabidopsis or Camelina, to which Pennycress is phylogenetically related or even with other members of the Thlaspideae tribe (Claver et al., 2017; Altendorf et al., 2019). Understanding the dynamics of seed TAG biosynthesis and of the incorporation of fatty acids to TAG is a necessary step to elucidate the biochemical nature of these differences and for the future improvement of the seed oil content or quality in Pennycress.

The RNA-Seq analysis was performed on five different maturation stages, covering the whole seed maturation process. A recent transcriptome analysis was reported in Pennycress in natural variants with differences in seed oil content (Arias et al., 2023). In their study, two early developmental stages that might correspond to our initial G stage and an even earlier stage were used. The analysis of the five different developmental stages in our work has allowed us to perform a complete study of the temporal pattern of gene expression during the whole process of seed maturation, from the earlier (G, GY), intermediate (YG), to the late (Y, M) ones. This is illustrated in the number of DEGs identified in each maturation stage comparison or the changes in the upregulated to downregulated ratios (**Figure 2**) as well as the evolution of DEGs in the GO analysis (**Figures 3**, **4**). In fact, many genes involved in fatty acid and lipid biosynthesis or lipid droplet formation showed sequential changes in their expression patterns as a result of the different processes occurring during seed maturation. Thus, genes involved in fatty acid biosynthesis like *TaFATA*, *TaFATB*, *TaKAS1*, *TaKAS2* or *TaLACS*, those encoding acyl-ACP carrier proteins (ACPs) involved in the transfer of acyl groups, or those encoding lipid transfer proteins (LTPs) like *TaLTP4*, *TaLTP5* and *TaLTP6*, were highly expressed at the early stages of seed maturation, decreasing in the latter ones (**Figures 5**, **6**). Other genes that showed high expression values at the G stage decreasing upon maturation, were those encoding photosynthetic proteins as well as other photosynthetic membrane formation or processes (**Figures 3**, **4**). This might be consistent with the loss of chlorophyll with seed maturation as seen in **Figure 1A** or the decrease in plastid lipids MGDG, DGDG or SQDG observed in **Figure 8**. In their study with early seed maturation stages, Arias et al. (2023) detected genes involved in photosynthesis among the most upregulated ones. Our data are consistent with this observation and suggest an important role of photosynthesis providing carbon and reducing power for fatty acid biosynthesis at the early stages of seed maturation. On the contrary, genes involved in lipid droplet formation, the final step of oil accumulation, like *TaOLE1*, *TaOLE2* and *TaOBAP1A*, peaked at the intermediate-late stages of seed maturation, YG or even Y, concomitant with the highest accumulation of TAG in the total lipid fractions (**Figures 8**, **9**).

Our lipidomic data showed not only that TAG levels increased (**Figure 8**), but also that the distribution of acyl groups in TAG varied with seed maturation. Thus, the LC-MS and HPTLC-MS data showed that TAG species containing 16:0, 18:1 or 18:2 acyl groups like 54:5, 56:3 or 58:5 were highly abundant at the initial G stage (**Figures 9A**, **10**), while upon seed maturation, VLCFAs containing TAG species like 60:4, containing 20:1, and particularly 62:4, containing 22:1, increased dramatically, particularly at the GY-Y stages, becoming the major TAG species in the total lipid fractions (**Figures 9A**, **10**). It is worth mentioning that the two different techniques used in this study, LC-MS and HPTLC-ESI-MS, identified the same TAG species with similar distribution changes upon seed maturation. Furthermore, these results confirmed our previous TLC-GC data (Claver et al., 2017) and those recently obtained using MS quadrupole analysis (Romsdahl et al., 2022). This high accumulation of TAG species containing VLCFAs was consistent with the expression of the *TaFAE1* elongase gene that increased from G to YG, up to 3-4 fold (**Figures 7** and **8**). On the other hand, the presence of TAG species containing 20:1 or 22:1 already at the G stage indicated that 22:1 was rapidly available for its incorporation to TAG at the early stages of seed maturation (**Figures 1**, **9A**) (Claver et al., 2017). Question arises which is the contribution of the different TAG biosynthetic pathways to the different content and acyl distribution of TAG observed in this study. The correlation between the lipidomic data and the expression analysis might help to answer this question.

**Figure 12:**
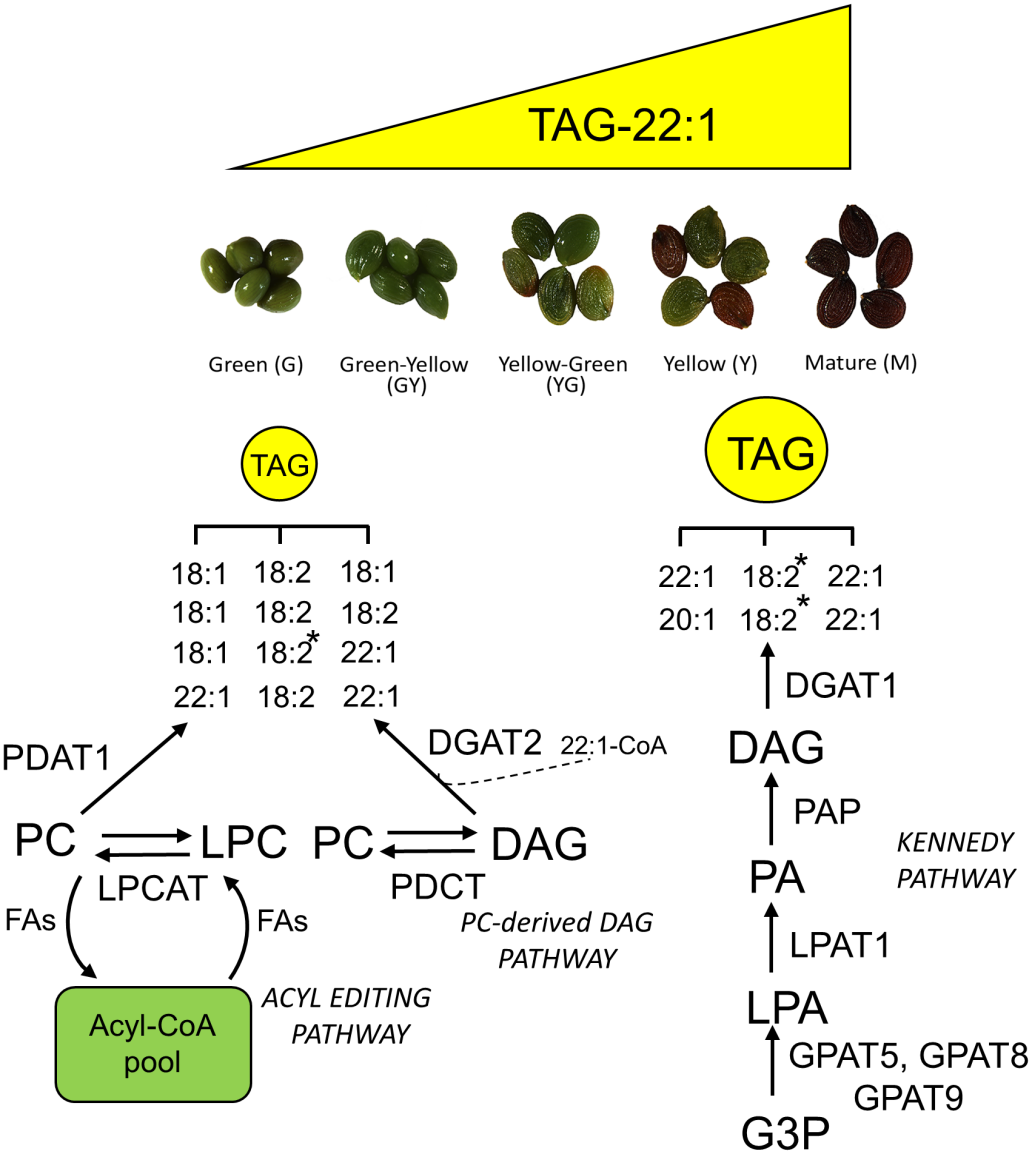
Schematic diagram showing a working model of the TAG biosynthesis pathway and the incorporation of erucic acid to TAG during Pennycress seed maturation. Lipid species abbreviations are as follows: DAG, diacylglycerol; G3P, glycerol-3-phosphate; LPA, lysophosphatidic acid; LPC, lysophosphatydilcholine; PA, phosphatidic acid; TAG, triacylglycerol. Enzyme abbreviations are as follows: DGAT, diacylglycerol acyltransferase; GPAT, glycerolphosphate acyltransferase; LPAT, lysophosphatidyl acyltransferase; LPCAT, lysophosphatidylcholine acyltransferase; PAP, phosphatidic acid phosphatase; PDAT, phospholipid-diacylglycerol acyltransferase; PDCT, phosphatidylcholine:diacylglycerol choline phosphotransferase. The asterisk at 18:2 indicates the TAG species in which the acyl position at *sn*-2 has been experimentally determined by MS^n^. The dashed line at DGAT2 suggest a possible role of this enzyme for the rapid incorporation of 22:1 to TAG at the early stages of seed maturation.

The expression of genes involved in TAG biosynthesis showed a complex temporal regulation pattern between pathways and between enzymes of the same pathway during Pennycress seed maturation. Thus, DGAT1 and DGAT2 are the main acyltransferases acting on the Kennedy pathway for the last acylation of DAG to produce TAG (Weselake et al., 2009; Li et al., 2012; Bates et al., 2013). Analysis of Arabidopsis mutants indicated that *At*DGAT1 was the major acyltransferase involved in TAG biosynthesis (Katavic et al., 1995; Regmi et al., 2020). The role of DGAT2 is less understood although it has been reported that specific *Bn*DGAT2 isoforms are involved in erucoyl-CoA incorporation to TAG in Brassica (Demski et al., 2019). In Pennycress, both RNA-Seq and qPCR analysis showed a complete opposite pattern of expression of both *TaDGAT* genes during seed maturation. Thus, *TaDGAT2* showed higher expression at the earlier stages, then decreasing in the later ones while *TaDGAT1* expression showed 3.5 to 4.5-fold increases from the G or GY to the Y and M stages, consistent with the increase in TAG levels and the detection of 62:4 as the major TAG species (**Figures 7**–**9**). As mentioned in the Results section, FPKM values of both *TaDGAT* genes indicated that *TaDGAT2* mRNA levels were more abundant than those from *TaDGAT1* at the G stage of maturation or similar at the GY one. This observation of higher expression of *TaDGAT2* at the early stages of seed development is consistent with previous observations in Tung (Shockey et al., 2006). These data suggested a coordination between both DGAT enzymes in which *Ta*DGAT1 would be responsible of the major acyltransferase activity through the Kennedy pathway during seed maturation and higher bulk TAG accumulation while *Ta*DGAT2 could participate in TAG biosynthesis at the earlier stages of seed development (**Figure 12**). This role of *Ta*DGAT enzymes, particularly *Ta*DGAT1, and the Kennedy pathway for the synthesis of the bulk TAG enriched in VLCFAs and particularly 22:1, would be supported by the expression pattern of the *TaLPAT* genes (*TaLPAT1*) or some *TaGPAT* genes (*TaGPAT5*, *TaGPAT8* and *TaGPAT9*), (**Figure 7**) whose expression pattern followed that of the *TaDGAT1* gene. Furthermore, the glycerolipid LC-MS analysis of species other than TAG also supported a relevant role of the Kennedy pathway, particularly from the intermediate YG to the mature stages of seed development. Thus, the DAG species detected, as well as the changes in their acyl group distribution during seed maturation, with higher presence of DAG species containing C16 and C18 fatty acids (like 34:2, 36:4 or 36:5) at the G stage, and further increase of DAG species like 40:3 or 40:4, containing 22:1, at the Y or M stages support that DAG could be acting as a reservoir of 20:1 and 22:1 for their incorporation to TAG through the Kennedy pathway during maturation of the Pennycress seed.

PC is also an important reservoir of acyl groups for their mobilization to TAG through the acyl-editing pathway or through PC-derived DAG/TAG biosynthetic pathways (Bates et al., 2013). In Arabidopsis (mostly containing 18:1 and 18:2 fatty acids esterified to TAG), it has been estimated that 40% of fatty acids in TAG were originated from the acyl editing pathway (Lu et al., 2009). In Crambe, a species accumulating VLCFAs, PDAT activity corresponded to a 10% of the DGAT one, particularly at the rapid oil accumulation stages (Furmanek et al., 2014). In Pennycress, PC content was always higher than that of DAG in all maturation stages (**Figure 8**). Furthermore, LC-MS analysis of PC showed species like 34:2, 36:3 or 36:4 (containing C16 and C18 fatty acids) as the major species detected in all seed maturation stages (**Figure 9C**). However, as occurred with DAG, PC species like 38:2 or 38:3, containing 20:1, or 40:2 and 40:3, containing 22.1, were also detected in all seed maturation stages, although in much lower amounts (**Figure 9C**). These species increased their relative content notably between the G and the GY/YG stages, coincident with the high increases in TAG biosynthesis (**Figure 8**). It is also true that, despite their increase and differently to what happened with DAG, these 20:1 and 22:1 containing PC species were never the major PC species in the Pennycress seed. These results contrasted with previous data from Romsdahl et al. (2022) that did not detect 22:1 in PC and low levels of these VLCFAs in DAG, contrasting with the high 22:1 levels in TAG. Nevertheless, our lipidomic data support a model in which a portion of the TAG detected in the Pennycress seed could be synthetized either through acyl-editing or PC-derived DAG/TAG biosynthesis. Again, this conclusion is further supported by the gene expression analysis. Thus, *TaPDAT1* mRNA levels showed higher expression at the G or GY maturation stages, later decreasing with seed maturation (**Figure 7**). Interestingly, the *TaLPCAT1* gene maintained its expression levels in all seed maturation stages, suggesting that the PC/LPC interconversion system was operative all-through seed maturation although the higher *TaPDAT1* expression levels at the initial maturation stages suggest that acyl-editing might contribute to TAG mostly at the beginning of seed maturation (**Figure 12**). This might be consistent with the higher presence of C16 and C18 fatty acids and particularly PUFAs in PC and TAG, which were higher at these stages (**Figure 9**). In addition, a specific contribution of PC-derived DAG/TAG biosynthesis in these initial stages cannot be precluded. Expression of the *TaPDCT* (*ROD1*) gene, encoding the enzyme that extracts the phosphate group from PC to produce DAG (Lu et al., 2009) showed higher expression levels at the G or GY stages of seed maturation, declining to undetectable levels in the rest of the stages (**Figure 7**). This result suggested that the PC-derived DAG/TAG biosynthetic pathway should be restricted to these initial stages of seed maturation, but not in mature seeds. *ROD1* mutants obtained in Pennycress did not show modifications in their TAG content when analyzed in mature seeds (Jarvis et al., 2021), consistent with our expression data. Interestingly, *TaDGAT2* expression values also showed a similar expression pattern, with higher expression at the G and GY stages (**Figure 7**). It is tempting to speculate that *Ta*DGAT2 might use this PC-derived DAG pool for TAG biosynthesis at the early stages of seed maturation while *Ta*DGAT1 should use *de novo* DAG for the bulk TAG accumulation, incorporating VLCFAs to TAG (**Figure 12**). In that sense, it was recently reported that in Arabidopsis, *At*PDAT1 and *At*DGAT2 used a different larger bulk of PC-derived DAG than that used by *At*DGAT1 (Regmi et al., 2020). The existence of these two DAG pools is consistent with previous analyses of acyl fluxes in soybean embryos (Bates et al., 2009). It is difficult to determine the size of these DAG pools and their modifications with seed maturation in Pennycress. HPTLC-MS/MS and MS^3^ spectra demonstrated that 18:2 was at *sn-2* position of the most abundant TAG species, independently of the acyl group esterified at the other two positions (**Figure 11** and **Table 1**). This observation is consistent with previous data in other Brassicaceae (Taylor et al., 1994). Crambe contains a 60% of 22:1 in TAG but only 10% of the *sn-2* positions of TAG were occupied by 22:1 (Li et al., 2012). This low proportion of 22:1 at *sn-2* has been attributed to low affinity of LPAT for the incorporation of VLCFAs to TAG (Taylor et al., 1994). This seems to be also the case of the *Ta*LPAT enzyme from Pennycress. Unfortunately, this *sn-2* signature does not allow to distinguish the origin of the DAG molecule in which the 3^rd^ acylation was performed.

In conclusion, our results support a model in which different pathways and different enzymes of the same pathway participate in TAG biosynthesis and acyl group incorporation and where these contributions may vary during Pennycress seed maturation. The Kennedy pathway might be acting during the whole process of seed maturation, showing higher DGAT1 activity with the higher TAG accumulation rates and higher erucic acid accumulation in TAG (**Figure 12**). In addition, our data suggest a specific contribution of the acyl-editing and PC-derived DAG/TAG pathways to TAG biosynthesis, particularly at the early stages of seed development (**Figure 12**). Metabolic flux analysis, which can be complicated in mature seed stages, together with a functional analysis of *DGAT* and *PDAT* mutants in Pennycress will help to clarify the specific contribution of each TAG biosynthetic pathways to seed oil biosynthesis in the Pennycress seed.

## Data availability statement

The datasets presented in this study can be found in online repositories. The names of the repository/repositories and accession number(s) can be found below: NCBI GEO (GSE256460), https://doi.org/10.20350/digitalCSIC/16109, http://hdl.handle.net/10261/346411.

## Author contributions

AC: Data curation, Formal analysis, Investigation, Writing – review & editing. ML: Data curation, Formal analysis, Investigation, Writing – review & editing. JE: Data curation, Formal analysis, Investigation, Writing – review & editing. MSc: Data curation, Formal analysis, Investigation, Methodology, Writing – review & editing. JJ: Data curation, Formal analysis, Investigation, Methodology, Writing – review & editing. MSa: Data curation, Formal analysis, Investigation, Methodology, Writing – review & editing. ML: Data curation, Formal analysis, Writing – review & editing, Funding acquisition, Investigation. RP: Writing – review & editing, Methodology, Supervision. CJ: Methodology, Writing – review & editing, Data curation, Formal analysis, Investigation. VC: Data curation, Formal analysis, Investigation, Methodology, Writing – review & editing, Supervision. MA: Data curation, Formal analysis, Investigation, Methodology, Supervision, Conceptualization, Funding acquisition, Project administration, Writing – original draft.

## Supplementary material

Supplementary Figure 3 Fatty acid distribution in the galactolipids MGDG, DGDG and SQDG **(A)** and phospholipids PG, PI, PS, PE and PA, **(B)** during Pennycress seed maturation. Values expressed in percentage of total lipids for each class. Seed maturation stages are indicated in the figure. Values presented are average of three determinations from two biological replicates; error bars represent SE. MGDG, monogalactosyldiacylglycerol; DGDG, digalactosyldiacylglycerol; SQDG, sulfoquinovosyldiacylglycerol; PG, phosphatidylglycerol; PI, phosphatidylinositol; PS, phosphatidyserine; PE, phosphatidylethanolamine; PA, phosphatidic acid.

Supplementary Table 2 Total reads, clean reads and quality parameters of the RNA-Seq data.

Supplementary Table 3 List of MRM transitions detected in the MS quadrupole analysis.

Supporting information 1 Standards and chemicals used in the HPTLS-ESI-MS analysis.

## References

[B1] Altendorf K. Isbell T. Wyse D. L. Anderson J. A. (2019). Significant variation of seed oil content, fatty acid profile, and seed weight in natural populations of filed Pennycress (Thlaspi arvense L.). Ind. Crop Prod. 129, 261–268. doi: 10.1016/j.indcrop.2018.11.054

[B2] Arias C. L. García Navarrete L. T. Mukundi E. Swanson T. Yang F. Hernández J.. (2023). metabolic and transcriptomic study of Pennycress natural variation identifies targets for oil improvement. Plant Biotechnol. J. 21 (9), 1887–1903. doi: 10.1111/pbi.14101 37335591 PMC10440992

[B3] Aznar-Moreno J. A. Denolf P. Van Audenhove K. De Bodt S. Engelen S. Fahy D.. (2015). Type 1 diacylglycerol acyltransferases of Brassica napus preferentially incorporate oleic acid into triacylglycerol. J. Exp. Bot. 66, 6497–6506. doi: 10.1093/jxb/erv363 26195728 PMC4588894

[B5] Bates P. D. Browse J. (2012). The significance if different diacylglycerol synthesis pathways on plant oil composition and bioengineering. Front. Plant Sci. 3. doi: 10.3389/fpls.2012.00147 PMC3387579 22783267

[B4] Bates P. D. Durret T. P. Ohlrogge J. Pollard M. (2009). Analysis of acyl fluxes through multiple pathways of triacylglycerol synthesis in developing soybean embryos. Plant Physiol. 150, 55–72. doi: 10.1104/pp.109.137737 19329563 PMC2675710

[B6] Bates P. D. Stymne S. Ohlrogge J. (2013). Biochemical pathways in seed oil synthesis. Curr. Op. Plant Biol. 16, 358–364. doi: 10.1016/j.pbi.2013.02.015 23529069

[B7] Baud S. Mendoza M. S. To A. Harscoët E. Lepiniec L. Dubreucq B. (2007). WRINKLED1 specifies the regulatory action of LEAFY COTYLEDON2 towards fatty acid metabolism during seed maturation in Arabidopsis. Plant J. 50, 825–838. doi: 10.1111/j.1365-313X.2007.03092.x 17419836

[B8] Bligh E. G. Dyer W. J. (1959). A rapid method of total lipid extraction and purification. Can. J. Biochem. Physiol. 37, 911–917. doi: 10.1139/y59-099 13671378

[B9] Buseman C. M. Tamura P. Sparks A. A. Baughman E. J. Maatta S. Zhao J.. (2006). Wounding stimulates the accumulation of glycerolipids containing oxophytodienoic acid and dinor-oxophytodienoic acid in Arabidopsis leaves. Plant Physiol. 142, 28–39. doi: 10.1104/pp.106.082115 16844834 PMC1557605

[B10] Cernac A. Benning C. (2004). WRINKLED1 encodes an AP2/EREB domain protein involved in the control of storage compound biosynthesis in Arabidopsis. Plant J. 40, 575–585. doi: 10.1111/j.1365-313X.2004.02235.x 15500472

[B11] Chen G. Snyder C. L. Greer M. S. Weselake R. J. (2011). Biology and biochemistry of plant phospholipases. Crit. Rev. Plant Sci. 30, 239–258. doi: 10.1080/07352689.2011.572033

[B12] Chopra R. Johnson E. B. Daniels E. McGinn M. Dorn K. M. Esfahanian M.. (2018). Translational genomics using Arabidopsis as a model enables the characterization of pennycress genes through forward and reverse genetics. Plant J. 96, 1093–1105. doi: 10.1111/tpj.14147 30394623

[B14] Claver A. de la Vega M. Rey-Giménez R. Luján M. A. Picorel R. López M. V.. (2020). Functional analysis of β-ketoacyl-CoA synthase from biofuel feedstock *Thlaspi arvense* reveals differences in the triacylglycerol biosynthetic pathway among Brassicaceae. Plant Mol. Biol. 104, 283–296. doi: 10.1007/s11103-020-01042-7 32740897

[B13] Claver A. Rey R. López M. V. Picorel R. Alfonso M. (2017). Identification of target genes and processes involved in erucic acid accumulation during seed development in the biodiesel feedstock Pennycress (*Thlaspi arvense* L.). J. Plant Physiol. 208, 7–16. doi: 10.1016/j.jplph.2016.10.011 27889523

[B15] Cubins J. A. Wells M. S. Frels K. Ott M. A. Forcella F. Johnsson G. A.. (2019). Management of Pennycress as a winter annual cash cover crop. A review. Agron. Sustain. Dev. 39, 1–11. doi: 10.1007/s13593-019-0592-0 30881486

[B16] Dahlqvist A. Ståhl U. Lenman m. Banas A. Lee M. Sandager L.. (2000). Phospholipid: diacylglycerol acyltransferase: an enzyme that catalyzes the acyl-CoA-independent formation of triacylglycerol in yeast and plants. Proc. Natl. Acad. Sci. U.S.A. 97, 6487–6492. doi: 10.1073/pnas.120067297 10829075 PMC18631

[B17] Demé B. Cataye C. Block M. A. Maréchal E. Jouhet J. (2014). Contribution of galactoglycerolipids to the 3-dimensional architecture of thylakoids. FASEB J. 28, 3373–3383. doi: 10.1096/fj.13-247395 24736411

[B18] Demski K. Jeppson S. Lager I. Mistak A. Jasieniecka-Gazarkiewicz K. Waleron M.. (2019). Isoforms of Acyl-CoA:diacylglycerol acyltransferase2 differ substantially in their specificities toward erucic acid. Plant Physiol. 181, 1468–1479. doi: 10.1104/pp.19.01129 31619508 PMC6878005

[B19] Dorn K. M. Fankhauser J. D. Wyse D. L. Marks M. D. (2013). *De novo* assembly of the Pennycress (*Thlaspi arvense*) transcriptome provides tools for the development of a winter cropland biodiesel feedstock. Plant J. 75, 1028–1038. doi: 10.1111/tpj.12267 23786378 PMC3824206

[B20] Dorn K. M. Fankhauser J. D. Wyse D. L. Marks M. D. (2015). A draft genome of field Pennycress (*Thlaspi arvense*) provides tools for the domestication of a new winter biofuel crop. DNA Res. 22, 121–131. doi: 10.1093/dnares/dsu045 25632110 PMC4401323

[B21] Fan J. Shonnard D. R. Kalnes T. N. Johnsen P. B. Rao S. (2013). A life cycle assessment of Pennycress (*Thlaspi arvense* L.) –derived jet fuel and diesel. Biomass Bioener. 55, 87–100. doi: 10.1016/j.biombioe.2012.12.040

[B22] Farese R. V. Jr Walther T. C. (2009). Lipid droplets finally get a little R-E-S-P-E-C-T. Cell 139, 855–860. doi: 10.1016/j.cell.2009.11.005 19945371 PMC3097139

[B23] Furmanek T. Demski K. Banas W. Haslam R. Napier J. Stymne S.. (2014). The utilization of the Acyl-CoA and the involvement PDAT and DGAT in the biosynthesis of erucic acid-rich tryacylglycerols in Crambe seed oil. Lipids 49, 327–333. doi: 10.1007/s11745-014-3886-7 24578031 PMC3964307

[B25] García Navarrete T. Arias C. Mukundi E. Alonso A. P. Grotewold E. (2022). Natural variation and improved genome annotation of the emerging biofuel crop field Pennycress (*Thlaspi arvense*). G3 genes/genomes/genetics 12, jkac084. doi: 10.1093/g3journal/jkac084 35416986 PMC9157065

[B24] Gasic K. Hernández A. Korban S. (2004). RNA extraction from different apple tissues rich in polyphenols and polysaccharides for cDNA library construction. Plant Mol Biol. Rep. 22, 437–438. doi: 10.1007/BF02772687

[B27] Geng Y. Guan Y. Qiong L. Lu S. An M. James M.. (2021). Genomic analysis of field pennycress (*Thlaspi arvense*) provides insights into mechanisms of adaptation to high elevation. BMC Biol. 19, 143. doi: 10.1186/s12915-021-01079-0 34294107 PMC8296595

[B26] Ghanevati M. Jaworski J. G. (2001). Active-site residues of a plant membrane-bound fatty acid elongase beta-ketoacyl- CoA- synthase, FAE1 KCS. Biochim. Biophys. Acta 1530, 77–85. doi: 10.1016/S1388-1981(00)00168-2 11341960

[B28] Guan R. Lager I. Li X. Stymne S. Zhu L.-H. (2014). Bottlenecks in erucic acid accumulation in genetically engineered ultrahigh erucic acid *Crambe abyssinica* . Plant Biotech. J. 12, 193–203. doi: 10.1111/pbi.12128 PMC4286110 24119222

[B29] Hsu F.-F. Turk J. (2010). Electrospray ionization multiple-stage linear ion-trap mass spectrometry for structural elucidation of triacylglycerols: Assignment of fatty acyl groups on the glycerol backbone and location of double bonds. J. Am. Soc Mass Spectrom. 21, 657–669. doi: 10.1016/j.jasms.2010.01.007 20171120 PMC2847672

[B31] Jarne C. Membrado L. Savirón M. Vela J. Orduna J. Garriga R.. (2021). Globotriaosylceramide-related biomarkers of fabry disease identified in plasma by high-performance thin-layer chromatography-densitometry-mass spectrometry. J. Chromatogr. A. 1638, 461895. doi: 10.1016/j.chroma.2021.461895 33477028

[B30] Jarne C. Savirón M. Lapieza M. P. Membrado L. Orduna J. Galbán J.. (2018). High-Performance thin-Layer chromatography coupled with electrospray ionization tandem mass spectrometry for identifying neutral lipids and sphingolipids in complex samples. J. AOAC Int. 101, 1993–2000. doi: 10.5740/jaoacint.17-0329 29571302

[B32] Jarvis B. A. Romsdahl T. B. McGinn M. Nazarenus T. J. Cahoon E. B. Chapman K. D.. (2021). CRISPR/Cas9-induced fad2 and rod1 mutations stacked with fae1 confer high oleic acid seed oil in Pennycress (*Thlaspi arvense L.*). Front. Plant Sci. 12. doi: 10.3389/fpls.2021.652319 PMC8100250 33968108

[B33] Johnston C. García-Navarrete L. T. Ortiz E. Romsdahl T. B. Guzha A. Chapman K. D.. (2022). Effective mechanisms for improving seed oil production in Pennycress (*Thlaspi arvense* L.) highlighted by integration of comparative metabolomics and transcriptomics. Front. Plant Sci. 13. doi: 10.3389/fpls.2022.943585 PMC9330397 35909773

[B34] Jouhet J. Lupette L. Clerc O. Magneschi L. Bedhomme M. Collin S.. (2017). LC-MS/MS versus TLC plus GC methods: Consistency of glycerolipid and fatty acid profiles in microalgae and higher plant cells and effect of nitrogen starvation. PloS One 12, e0182423. doi: 10.1371/journal.pone.0182423 28771624 PMC5542700

[B36] Katavic V. Friesen W. Barton D. L. Gossen K. K. Giblin E. M. Luciw T.. (2001). Improving erucic acid content in rapeseed through biotechnology: What can the *Arabidopsis FAE1* and the yeast *SLC1-1* genes contribute. Crop Sci. 41, 739–747. doi: 10.2135/cropsci2001.413739x

[B35] Katavic V. Reed D. W. Taylor D. C. Giblin E. M. Barton D. L. Zou J.-T.. (1995). Alteration of seed fatty acid compostition by en ethylmethanesulfonate-induced mutation in Arabidopsis thaliana affecting diacylglycerol acyltransferase activity. Plant Physiol. 108, 399–409. doi: 10.1104/pp.108.1.399 7784510 PMC157347

[B37] Kim H. U. Huang A. H. (2004). Plastid lysophosphatidyl acyltransferase is essential for embryo development in Arabidopsis. Plant Physiol. 134, 1206–1216. doi: 10.1104/pp.103.035832 14976237 PMC389945

[B38] Kim H. U. Li Y. Huang A. H. (2005). Ubiquitous and endoplasmic reticulum lysophosphatidyl acyltransferase, LPAT2, is essential for female, but not male gametophyte development in Arabidopsis. Plant Cell 17, 1073–1089. doi: 10.1105/tpc.104.030403 15772283 PMC1087987

[B39] Lager I. Lindberg Yilmaz J. Zhou X. R. Jasieniecka K. Kazachkov M. Wang P.. (2013). Plant Acyl-CoA:lisophosphatidylcholine acyltransferases (LPCATs) have different specificities in their forward and reverse reactions. J. Biol. Chem. 288, 36902–36914. doi: 10.1074/jbc.M113.521815 24189065 PMC3873549

[B40] Li Y. H. Beisson F. Pollard M. Ohlrogge J. (2006). Oil content of Arabidopsis seeds: the influence of seed anatomy: light and plant to plant variation. Phytochem. 67, 904–915. doi: 10.1016/j.phytochem.2006.02.015 16600316

[B42] Li Z. van Loo E. N. Gruber J. Fan J. Guan R. Frentzen M.. (2012). Development of ultra-high erucic acid oil in the industrial oil crop Crambe abyssinica. Plant Biotechnol. J. 10, 862–870. doi: 10.1111/j.1467-7652.2012.00709.x 22642539

[B41] Li R. Yu K. Hildebrand D. F. (2010). *DGAT1*, *DGAT2* and *PDAT* expression in seeds and other tissues of epoxy and hidroxy fatty acid accumulating plants. Lipids 45, 145–157. doi: 10.1007/s11745-010-3385-4 20101470

[B43] Li-Beisson Y. Shorrosh B. Beisson F. Andersson M. X. Arondel V. Bates P. D.. (2013). Acyl-lipid metabolism. Arabidopsis book 11, 161. doi: 10.1199/tab.0161 PMC3563272 23505340

[B44] Livak K. J. Schmittgen T. D. (2001). Analysis of relative gene expression data using real-time PCR and the 2(-delta Delta C(T) method. Methods 25, 402–408. doi: 10.1006/meth.2001.1262 11846609

[B45] López M. V. de la Vega M. Gracia R. Claver A. Alfonso M. (2021). Agronomic potential of two European Pennycress accessions as a Winter crop under Mediterranean conditions. Ind. Crop Prod. 159, 113107. doi: 10.1016/j.indcrop.2020.113107

[B46] Lu C. Xin Z. Ren Z. Miquel M. (2009). An enzyme regulating triacylglycerol composition is encoded by the ROD1 gene of Arabidopsis. Proc. Natl Acad. Sci. U.S.A. 106, 18837–18842. doi: 10.1073/pnas.0908848106 19833868 PMC2774007

[B47] McGinn M. Phippen W. B. Chopra R. Bansal S. Jarvis B. A. Phippen M. E.. (2019). Molecular tools enabling Pennycress (*Thlaspi arvense*) as a model plant and oilseed cash cover crop. Plant Biotechnol. J. 17 (4), 776–788. doi: 10.1111/pbi.13014 30230695 PMC6419581

[B48] Men X. Shi J. Liang W. Zhang Q. Lian G. Quan S.. (2017). Glycerol-3-Phosphate Acyltransferase 3 (OsGPAT3) is required for anther development and male fertility in rice. J. Exp. Bot. 68 (3), 513–526. doi: 10.1093/jxb/erw445 28082511 PMC6055571

[B49] Mortazavi A. Williams B. A. McCue K. Chaeffer L. Wold B. (2008). Mapping and quantifying mammalian transcriptomes by RNA-Seq. Nat. Methods 5, 621–628. doi: 10.1038/nmeth.1226 18516045 PMC13303166

[B51] Moser B. R. (2012). Biodiesel from alternative oilseed feedstocks: Camelina and filed Pennycress. Biofuels 3, 193–209. doi: 10.4155/bfs.12.6

[B50] Moser B. R. Knothe G. Vaughn S. F. Isbell T. A. (2009). Production and evaluation of biodiesel from field Pennycress (*Thlaspi arvense* L.) oil. Energy Fuels 23, 4149–4155. doi: 10.1021/ef900337g

[B52] Nunn A. Rodríguez-Arévalo I. Tandukar Z. Frels K. Contreras-Garrido A. Carbonell-Bejerano P.. (2022). Chromosome-level *Thlaspi arvense* genome provides new tools for translational research and for a newly domesticated cash cover crop of the cooler climates. Plant Biotechnol. J. 20 (5), 944–963. doi: 10.1111/pbi.13775 34990041 PMC9055812

[B53] Ohlrroge J. Browse J. (1995). Lipid biosynthesis. Plant Cell 7, 957–970. doi: 10.1105/tpc.7.7.957 7640528 PMC160893

[B55] Rainteau D. Humbert L. Delage E. Vergnolle C. Cantrel C. Maubert M.A.. (2012). Acyl chains of phospholipase D transphosphatidylation products in Arabidopsis cells: A study using multiple reaction monitoring mass spectrometry. PloS One 7, e41985. doi: 10.1371/journal.pone.0041985 22848682 PMC3405027

[B54] Ramaley L. Herrera L. C. Melanson J. E. (2015). Quantitative analysis of TAG in oils using lithium cationization and direct-infusion ESI tandem mass spectrometry. J. Am. Oil Chem. Soc. 92, 323–334. doi: 10.1007/s11746-015-2604-9

[B56] Regmi A. Shockey J. Kotapati H.-K. Bates P. D. (2020). Oil-producing metabolons containing DGAT1 use separate substrate pools from those containing DGAT2 or PDAT. Plant Physiol. 184, 720–737. doi: 10.1104/pp.20.00461 32732347 PMC7536707

[B57] Romsdahl T. B. Cocuron J.-C. Pearson M. J. Alonso A. P. Chapman K. D. (2022). A lipidomics platform to analyze the fatty acid compositions of non-polar and polar lipid molecular species from plant tissues: Examples from developing seeds and seedlings of Pennycress (*Thlaspi arvense*). Front. Plant Sci. 13. doi: 10.3389/fpls.2022.1038161/full PMC9682148 36438089

[B58] Routaboul J. M. Benning C. Bechtold N. Caboche M. Lepiniec L. (1999). The TAG1 locus of Arabidopsis encodes for a diacylglycerol acyltransferase. Plant Physiol. Biochem. 37, 831–840. doi: 10.1016/S0981-9428(99)00115-1 10580283

[B59] Sancho-Albero M. Jarne C. Savirón M. Martín-Duque P. Membrado L. Cebolla V. L.. (2022). High-Performance thin-Layer chromatography-Densitometry-Tandem ESI-MS to evaluate phospholipid content in exosomes of cancer cells. Int. J. Mol. Sci. 23, 1150–1164. doi: 10.3390/ijms23031150 35163074 PMC8835402

[B60] Sedbrook J. C. Phippen W. B. Marks M. D. (2014). New approaches to facilitate rapid domestication of a wild plant to an oilseed crop: example Pennycress (*Thlaspi arvense* L.). Plant Sci. 227, 122–132. doi: 10.1016/j.plantsci.2014.07.008 25219314

[B61] Shockey J. M. Gidda S. K. Chapital D. C. Kuan J.-C. Dhanoa P. K. Bland J. M.. (2006). Tung tree DGAT1 and DGAT2 havew nonredundant function in triaacylglycerol biosynthesis and are localized to different subdomains of the endoplasmic reticulum. Plant Cell 18, 2294–2313. doi: 10.1105/tpc.106.043695 16920778 PMC1560902

[B62] Shockey J. Regmi A. Cotton K. Adhikari N. Browse J. Bates P. D. (2016). Identification of Arabidopsis GPAT9 (At5g60620) as an essential gene involved in triacylglycerol biosynthesis. Plant Physiol. 170, 163–179. doi: 10.1104/pp.15.01563 26586834 PMC4704598

[B63] Stymne S. Stobart A. K. (1984). Evidence of the reversibility of the acyl-coA-lysophosphatidylcholine acyltransferase in microsomal preparations from developing safflower (*Carthamus-tinctorius* L.) cotyledons and rat liver. Biochem. J. 223, 305–314. doi: 10.1042/bj2230305 6497849 PMC1144301

[B64] Sun X. Pang H. Guo H. Yan Q. Hang Y. (2013). Evolutionary pattern of the *FAE1* gene in *Brassicaceae* and its correlation with the erucic acid trait. PloS One 8, 1–12. doi: 10.1371/journal.pone.0083535 PMC3865303 24358289

[B65] Taylor D. C. Mackenzie S. L. McCurdy A. R. McVetty P. B. Giblin E. M. Pass E. W.. (1994). Stereospecific analyses of seed triacylglycerols from high-erucic acid brassicaceae: deletion of erucic acid at the *sn-2* position in *Brassica oleracea* L. genotypes. J. Am. Chem. Soc 71, 163–167. doi: 10.1007/BF02541551

[B66] Tsogtbaatar E. Cocuron J. C. CorChado Sonera M. Alonso A. P. (2015). Metabolite fingerprinting of Pennycress (*Thlaspi arvense L.*) embryos to assess active pathways during oil synthesis. J. Exp. Bot. 66, 4267–4277. doi: 10.1093/jxb/erv020 25711705 PMC4493779

[B70] Wang L. Shen W. Kazachkov M. Chen G. Chen Q. Carlsson A. S.. (2012). Metabolic interactions between the lands Cycle and the Kennedy pathway of glycerolipid synthesis in *Arabidopsis* developing seeds. Plant Cell 24, 4652–4669. doi: 10.1105/tpc.112.104604 23150634 PMC3531858

[B71] Weselake R. J. Taylor D. C. Rahman M. H. Shah S. Laroche A. McVetty P. B. E.. (2009). Increasing the flow of carbon into seed oil. Biotechnol. Adv. 27, 866–878. doi: 10.1016/j.biotechadv.2009.07.001 19625012

[B72] Xu J. Carlsson A. Francis T. Zhang M. Hoffmann T. Giblin M.. (2012). Tricaylglicerol synthesis by PDAT1 in the absence of DGAT1 activity is dependent on re-acylation of LPC by LPCAT2. BMC Plant Biol. 12, 4. doi: 10.1186/1471-2229-12-4 22233193 PMC3310826

[B67] Yang W. Pollard M. Li-Beisson Y. Beisson F. Feig M. Ohlrogge J. (2010). A distinct type of glycerol-3-phosphate acyltransferase with sn -2 preference and phosphatase activity producing 2-monoacylglycerol. Proc. Natl. Acad. Sci. U.S.A. 107, 12040–12045. doi: 10.1073/pnas.0914149107 20551224 PMC2900678

[B73] Yu G. Wang L. G. Han Y. He Q. Y. (2012). ClusterProfiler: an R package for comparing biological themes among gene clusters. OMICS 16, 284–287. doi: 10.1089/omi.2011.0118 22455463 PMC3339379

[B68] Zhang M. Fan J. Taylor D. C. Ohlrogge J. B. (2009). DGAT1 and PDAT1 acyltransferases have overlapping functions in Arabidopsis triacylglycerol biosynthesis and are essential for normal pollen and seed development. Plant Cell 21, 3885–3901. doi: 10.1105/tpc.109.071795 20040537 PMC2814504

[B69] Zheng Z. Xia Q. Dauk M. Shen W. Selvaraj G. Zou J. (2003). Arabidopsis *AtGPAT1*, a member of the membrane-bound glycerol-3-phosphate acyltransferase gene family, is essential for tapetum differentiation and male fertility. Plant Cell 15, 1872–1887. doi: 10.1105/tpc.012427 12897259 PMC167176

