# Supplementary material for: Transcriptomic and lipidomic analysis of the differential pathway contribution to the incorporation of erucic acid to triacylglycerol during Pennycress seed maturation
Source: Front Plant Sci. 2024 Apr 26;15:1386023. doi: 10.3389/fpls.2024.1386023 (PMC11082276; doi:10.3389/fpls.2024.1386023)
Supplement: Supplementary Figure 3 — Fatty acid distribution in the galactolipids MGDG, DGDG and SQDG (A) and phospholipids PG, PI, PS, PE and PA, (B) during Pennycress seed maturation. Values expressed in percentage of total lipids for each class. Seed maturation stages are indicated in the figure. Values presented are average of three determinations from two biological replicates; error bars represent SE. MGDG, monogalactosyldiacylglycerol; DGDG, digalactosyldiacylglycerol; SQDG, sulfoquinovosyldiacylglycerol; PG, phosphatidylglycerol; PI, phosphatidylinositol; PS, phosphatidyserine; PE, phosphatidylethanolamine; PA, phosphatidic acid. [file Table_1.docx]

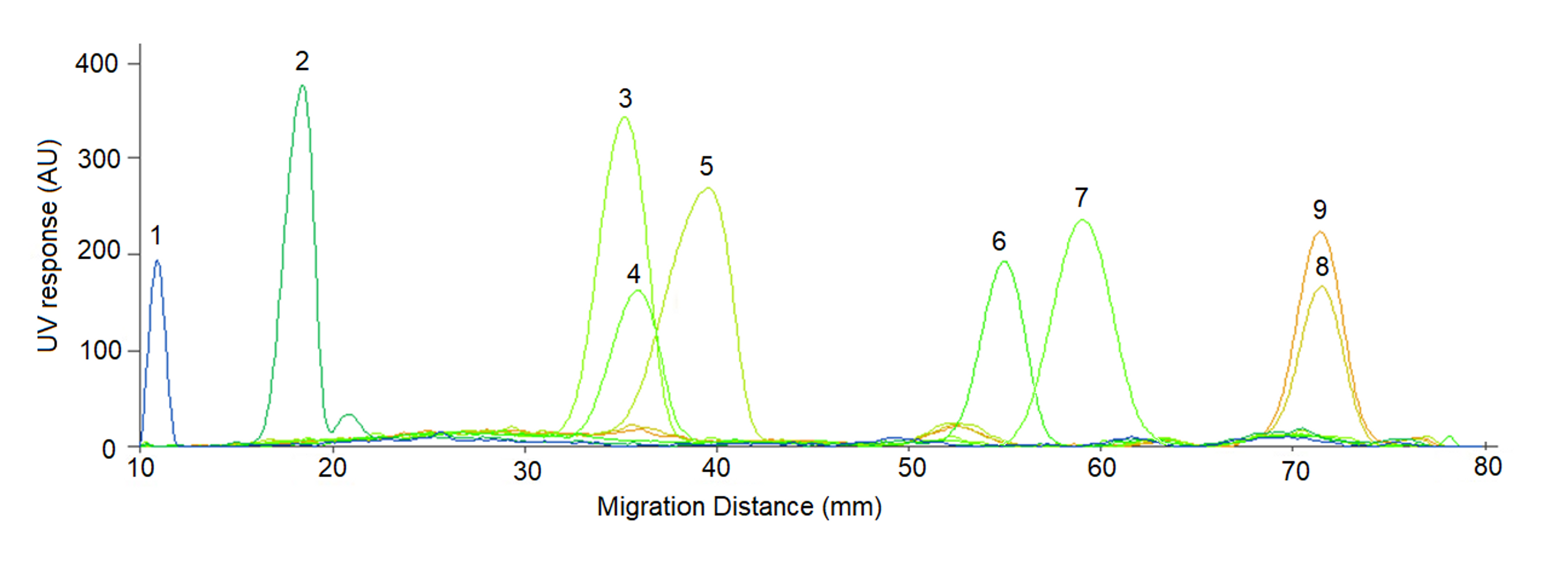


**Fig. S1**. HPTLC chromatograms of standards at UV 190 nm: pic1: 1-Oleoyl-rac-glycerol, pic 2: 1,2-dioleoyl-*rac*-glycerol, pic 3: linoleic acid, pic 4: oleic acid, pic 5: erucic acid, pic 6: glyceryl trioleate, pic 7: methyl oleate, pic 8: cholesteryl stearate, and pic 9: cholesteryl oleate.
