# Supplementary material for: Transcriptomic and lipidomic analysis of the differential pathway contribution to the incorporation of erucic acid to triacylglycerol during Pennycress seed maturation
Source: Front Plant Sci. 2024 Apr 26;15:1386023. doi: 10.3389/fpls.2024.1386023 (PMC11082276; doi:10.3389/fpls.2024.1386023)
Supplement: Supplementary Table 2 — Total reads, clean reads and quality parameters of the RNA-Seq data. [file Table_2.docx]

**Fig. S2.** HPTLC chromatograms corresponding to samples at the different maturation stages detected at UV 190 nm: a) GREEN, b) GREENYELLOW, c) YELLOWGREEN; d) YELLOW, and e) MATURE.
