# Supplementary material for: Transcriptomic and lipidomic analysis of the differential pathway contribution to the incorporation of erucic acid to triacylglycerol during Pennycress seed maturation
Source: Front Plant Sci. 2024 Apr 26;15:1386023. doi: 10.3389/fpls.2024.1386023 (PMC11082276; doi:10.3389/fpls.2024.1386023)
Supplement: Supporting information 1 — Standards and chemicals used in the HPTLS-ESI-MS analysis. [file Table_4.docx]

**Standards and chemicals used in HPTLC analysis.**

1-Oleoyl-rac-glycerol (C18:1, ≥ 99 %, 111-03-5 CAS); 1,2-dioleoyl-*rac*-glycerol (C18:1/C18:1, ≥ 97 %, 2442-61-7 CAS); glyceryl trioleate (C18:1/C18:1/C18:1, >99 %, 22-32-7 CAS); oleic acid (C18:1), ≥ 99 %, 112-80-1 CAS); linoleic acid (C18:2, ≥ 99 %, 60-33-3 CAS); erucic acid (C22:1, ≥ 99 %, 112-86-7 CAS); methyl oleate (99 %, 112-62-9 CAS); cholesteryl oleate ( ≥ 98 %, 303-43-5 CAS); cholesteryl stearate ( 96 %, 35602-69-8 CAS),were purchased from Sigma-Aldrich (Madrid, Spain). Notation adopted for the identity of a molecular species was (x:y) where x is the carbon number of the fatty acid-chains of the molecule; and y corresponds to the total number of double bonds. HPLC-grade *n*-heptane (C7, 99.9 %), tert-butyl methyl ether (MTBE, 99.9 %), glacial acetic acid (AcH, 99.8 %), methanol (MeOH, 99.9 %) dichloromethane (DCM, 99.9 %) and tetrahydrofuran (THF, 99.9 %, without stabilizer) were purchased from PanReac (Barcelona, Spain). LC/MS- grade MeOH was purchased from Fisher (UK). HPTLC silica gel 60 plates (20 × 10 cm) without fluorescence indicator were employed, from Merck (Darmstadt, Germany).
