# Supplementary material for: Transcriptomic and lipidomic analysis of the differential pathway contribution to the incorporation of erucic acid to triacylglycerol during Pennycress seed maturation
Source: Front Plant Sci. 2024 Apr 26;15:1386023. doi: 10.3389/fpls.2024.1386023 (PMC11082276; doi:10.3389/fpls.2024.1386023)
Supplement: Supplementary file 5 [file Table_5.docx]

Table S1. List of qPCR primers used in this study

________________________________________________

*TaFAE1:*

qF_TaFAE1 [ACGGAACGCGGCAAGCGAT](https://www.thermofisher.com/order/catalog/product/10336022?tsid=Email_POE_OC_OrderConfirm%20%0D%20_SKULINK)

qR_TaFAE1 [CCAGACCGCTCCTGAATCTTCCT](https://www.thermofisher.com/order/catalog/product/10336022?tsid=Email_POE_OC_OrderConfirm%20%0D%20_SKULINK)

*TaFAD2:*

qF_FAD2 TTCACGCTCGGAGAACTGAAG

qR_FAD2 GATAAGGTAGGAGAAAGAGCG

*TaDGAT1:*

qF_DGAT1 GTACCGATGCTTTCCCATCAGA

qR_DGAT1 GCCAAATTCGCTGTTCCCTG

*TaDGAT2*

qF_DGAT2 TCTATCGCTCCTGGTCTTTTGTC

qR_DGAT2 TACCTAGCGAGCTTACGACCG

*TaPDCT*

[qF_PDCT1 CGAGGCATTCTTGGTTACTC](https://www.thermofisher.com/order/catalog/product/10336022?tsid=Email_POE_OC_OrderConfirm%20%0D%20_SKULINK)

[qR_PDCT1 CCCGAGTAGAAGAGGAAGAA](https://www.thermofisher.com/order/catalog/product/10336022?tsid=Email_POE_OC_OrderConfirm%20%0D%20_SKULINK)

*TaPDAT1*

[qF_TaPDAT1 TGAGCTCTGGGAAGGTAAA](https://www.thermofisher.com/order/catalog/product/10336022?tsid=Email_POE_OC_OrderConfirm%20%0D%20_SKULINK)

[qR_TaPDAT1 GTGTTCCACCCAACATAGAG](https://www.thermofisher.com/order/catalog/product/10336022?tsid=Email_POE_OC_OrderConfirm%20%0D%20_SKULINK)

*TaPDAT2*

[qF_TaPDAT2](https://www.thermofisher.com/order/catalog/product/10336022?tsid=Email_POE_OC_OrderConfirm%20%0D%20_SKULINK) CGAGCTCTGGGAAGGTCGG

[qR_TaPDAT2](https://www.thermofisher.com/order/catalog/product/10336022?tsid=Email_POE_OC_OrderConfirm%20%0D%20_SKULINK) CTTAATATTTTGTTTACC

*TaLPCAT*

[qF_TaLPCAT GAAATGGGACCGTGCTAAGA](https://www.thermofisher.com/order/catalog/product/10336022?tsid=Email_POE_OC_OrderConfirm%20%0D%20_SKULINK)

[qR_TaLPCAT CCAGGTGCTGACTTGTATGT](https://www.thermofisher.com/order/catalog/product/10336022?tsid=Email_POE_OC_OrderConfirm%20%0D%20_SKULINK)

*TaLPAT1*

[qF_TaLPAT1: AAGGATGGTCGGTTAGGT](https://www.thermofisher.com/order/catalog/product/10336022?tsid=Email_POE_OC_OrderConfirm%20%0D%20_SKULINK)

[qR_TaLPAT1: TTTGGCCTGTTCCCATTAG](https://www.thermofisher.com/order/catalog/product/10336022?tsid=Email_POE_OC_OrderConfirm%20%0D%20_SKULINK)

*TaWRI1:*

qF_WRI1 GGAACTGGAGGAAATGCAGAG

qR_WRI1 GAGCCTCCCATCTTCCGTT

*TaOLE1*

OLE1FW: GAGCACCCACAGGGTTCA

OLE1RV: CATGTTCTCCACCAGTATGTTGCT

*TaOLE2*

OLE2FW: GCACTTGCAATTTCAGTCACC

OLE2RV: CACGTTCAGGCATCATGC

*TaOBAP1a*

OBAP1FW: CCTAATATCATGATGGCCGT

OBAP1aRV: CTCTTGGAACGGACTCGAC

______________________________________________________
