# Supplementary material for: Transcriptomic and lipidomic analysis of the differential pathway contribution to the incorporation of erucic acid to triacylglycerol during Pennycress seed maturation
Source: Front Plant Sci. 2024 Apr 26;15:1386023. doi: 10.3389/fpls.2024.1386023 (PMC11082276; doi:10.3389/fpls.2024.1386023)
Supplement: Supplementary file 8 [file Table_8.docx]

**Supplementary Table 4.** Percentages of TAG peak in UV (190 nm)-HPTLC chromatograms of oil samples, and intra- and inter-plate HPTLC repeatability results for the separated TAG peaks (expressed in Area counts).

| **Maduration stage/batch** | **TAG%** | **Intraplate Repeatability** | | | | **Interplate Repeatability** | | | |
| --- | --- | --- | --- | --- | --- | --- | --- | --- | --- |
|  |  | **Mean** | **SD** | **RSD%** | **CV** | **Mean** | **SD** | **RSD%** | **CV** |
| G/1 | 91.1% | 9442.71 | 389.06 | 4.12 | 359.84 | 9377.38 | 297.94 | 3.18 | 180.06 |
| G/2 | 88.1% | 12284.57 | 370.23 | 3.01 | 342.42 | 12600.08 | 500.08 | 3.97 | 302.22 |
| GY/1 | 92.7% | 15556.71 | 847.40 | 5.45 | 783.74 | 15968.54 | 774.08 | 4.85 | 467.81 |
| GY/2 | 91.9% | 14830.29 | 600.25 | 4.05 | 555.15 | 17465.08 | 510.93 | 2.93 | 308.78 |
| YG/1 | 95.3% | 14830.29 | 600.25 | 4.05 | 555.15 | 15787.08 | 1241.20 | 7.86 | 750.12 |
| YG/2 | 95.0% | 16867.14 | 385.73 | 2.29 | 356.75 | 17140.92 | 642.26 | 3.75 | 388.15 |
| Y/1 | 95.8% | 15531.14 | 403.41 | 2.60 | 373.10 | 16668.62 | 1408.25 | 8.45 | 851.07 |
| Y/2 | 95.5% | 10920.29 | 284.65 | 2.61 | 263.26 | 11932.92 | 1367.02 | 11.46 | 826.15 |
| M/1 | 94.0% | 15779.43 | 402.49 | 2.55 | 372.25 | 17312.85 | 2184.14 | 12.62 | 1319.98 |
